# Mechanism of activation and biased signaling in complement receptor C5aR1

**DOI:** 10.1038/s41422-023-00779-2

**Published:** 2023-02-17

**Authors:** Yuying Feng, Chang Zhao, Yue Deng, Heli Wang, Liang Ma, Sicen Liu, Xiaowen Tian, Bo Wang, Yan Bin, Peipei Chen, Wei Yan, Ping Fu, Zhenhua Shao

**Affiliations:** Division of Nephrology and Kidney Research Institute, State Key Laboratory of Biotherapy and Cancer Center, West China Hospital, Sichuan University, Chengdu, Sichuan China

**Keywords:** Cryoelectron microscopy, Cell signalling

## Abstract

The complement system plays an important role in the innate immune response to invading pathogens. The complement fragment C5a is one of its important effector components and exerts diverse physiological functions through activation of the C5a receptor 1 (C5aR1) and associated downstream G protein and β-arrestin signaling pathways. Dysfunction of the C5a-C5aR1 axis is linked to numerous inflammatory and immune-mediated diseases, but the structural basis for activation and biased signaling of C5aR1 remains elusive. Here, we present cryo-electron microscopy structures of the activated wild-type C5aR1–G_i_ protein complex bound to each of the following: C5a, the hexapeptidic agonist C5a^pep^, and the G protein-biased agonist BM213. The structures reveal the landscape of the C5a–C5aR1 interaction as well as a common motif for the recognition of diverse orthosteric ligands. Moreover, combined with mutagenesis studies and cell-based pharmacological assays, we deciphered a framework for biased signaling using different peptide analogs and provided insight into the activation mechanism of C5aR1 by solving the structure of C5aR1^I116A^ mutant–G_i_ signaling activation complex induced by C089, which exerts antagonism on wild-type C5aR1. In addition, unusual conformational changes in the intracellular end of transmembrane domain 7 and helix 8 upon agonist binding suggest a differential signal transduction process. Collectively, our study provides mechanistic understanding into the ligand recognition, biased signaling modulation, activation, and G_i_ protein coupling of C5aR1, which may facilitate the future design of therapeutic agents.

## Introduction

The complement system is an important component of the innate immune response to infectious pathogens. Complement activation by foreign agents leads to the production of proinflammatory factors, which can interact with the innate and adaptive immune systems.^1^ The upregulation or downregulation of the complement system is associated with several acute and chronic diseases including sepsis, asthma, anti-neutrophil cytoplasmic autoantibody-associated vasculitis, cancer, and coronavirus disease 2019.^2–6^

The 74-amino-acid complement fragment C5a, which is a key proinflammatory mediator and an anaphylatoxin, is generated during the complement cascade by the cleavage of C5.^4^ C5a binds to the C5a receptor (C5aR) 1, a prototypical member of the G protein-coupled receptor (GPCR) superfamily, leading to recruitment of the G protein subtypes G_i_, G_q_, and G_16_ and β-arrestins^7–10^ and, ultimately, to numerous cell-specific responses including chemotaxis, phagocytosis, and cytokine release.^11^ During the SARS-CoV-2 pandemic, clinical investigations revealed that patients with severe illness exhibited widespread activation of the C5a-C5aR1 axis.^2^ C5a can also bind to C5aR2, an atypical GPCR that recruits only β-arrestins and lacks any detectable functional coupling to G proteins,^12^ leading to pleiotropic immune-dampening functions.^13,14^

The recruitment of β-arrestins to C5aR1 results primarily in the termination of G protein signaling via inducing desensitization and internalization of the receptor.^12^ A new paradigm that has emerged during the past decades is that β-arrestins can mediate downstream signaling either on their own or by interacting with G proteins.^15,16^ Additionally, C5a-induced β-arrestin-mediated cell-specific responses such as chemotaxis and cytokine release have been proposed.^16^ Therefore, detailed understanding of the activation mechanism and biased signaling of the C5a-C5aR1 axis may facilitate the elucidation of complement-mediated pathophysiology.

In the present study, we performed structural and pharmacological analyses of the complement cascade. We solved cryo-electron microscopy (cryo-EM) structures of activated wild-type C5aR1–G_i_ protein bound to each of C5a, the hexapeptidic agonist C5a^pep^ (which has functional bias in terms of trafficking and cellular outcomes^17^), and the G protein-biased agonist BM213. We also solved the structure of the C5aR1 (I116^3.32^A) mutant–G_i_ protein complex bound to the ligand C089. The structures revealed a three-site binding mode of C5a to C5aR1 and a mechanism for diverse ligand recognition. Together with functional assay results, our data elucidate the signaling features of β-arrestin-biased ligands and identifies a “zipper-like” hydrophobic interface required for conformational changes in the receptor.

## Results

### Three-site binding mode of C5a–C5aR1 complexes revealed by cryo-EM

**Fig. 1:**
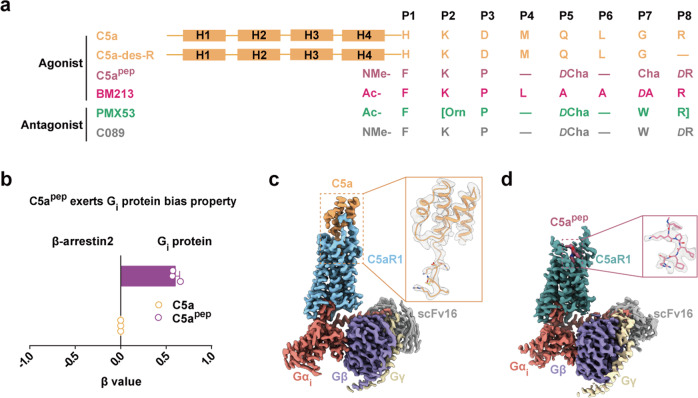
Representatives of C5aR1 ligands, as well as overall structures of C5aR1–Gi complex bound to C5a or C5a^pep^. **a** Sequence alignment of agonists and antagonists of C5aR1 used in this study. The chemical structures of non-natural amino acids are shown in Supplementary information, Fig. S1a. The peptide cyclization is shown with “[]” and d-amino acid is indicated as “*D*”. **b** C5a^pep^ induces G_i_ protein-biased signaling compared to C5a. Bias factor of C5a^pep^ was derived from curve fit parameters from Supplementary information, Fig. S1b and was calculated using the endogenous agonist C5a as the reference. Data are presented as the means ± SEM of three independent experiments performed in triplicate. **c**, **d** Cryo-EM maps of C5a-bound (**c**) and C5a^pep^-bound (**d**) C5aR1**–**G_i_ complexes. Sandy brown, C5a; pale violet red, C5a^pep^; light sky blue, C5a-bound C5aR1; cadet blue, C5a^pep^-bound C5aR1; salmon, Gα_i_; slate blue, Gβ; wheat, Gγ; dark gray, scFv16.

Human C5a is composed of four α-helix bundles (residues 1–64) and a C-terminal tail (residues 65–74). A series of peptide ligands targeting C5aR1 are derived from the C-terminal tail of C5a, which has been identified as an important active pharmacophore (Fig. 1a; Supplementary information, Fig. S1a).^18,19^ Among these, C5a^pep^ possesses a high binding affinity for C5aR1; it also has a biased functional efficacy toward G_i_ protein signaling, in contrast to the functionally unbiased C5a (Fig. 1b; Supplementary information, Fig. S1b).^17^ To understand the mechanism of C5aR1 signal induction in response to C5a or C5a^pep^, we first determined the cryo-EM structures of active C5aR1–G_i_ protein complex bound to either C5a or C5a^pep^ at resolutions of 2.9 Å or 3.2 Å, respectively (Fig. 1c, d; Supplementary information, Figs. S2, S3 and Table S1). The structures reveal that G_i_ protein binds C5aR1 in a similar manner to that of the μ-opioid receptor and the cannabinoid receptor 1 complexes, indicating that the agonist-bound C5aR1 signaling complex is in an active conformation (Supplementary information, Fig. S4).^20,21^

**Fig. 2:**
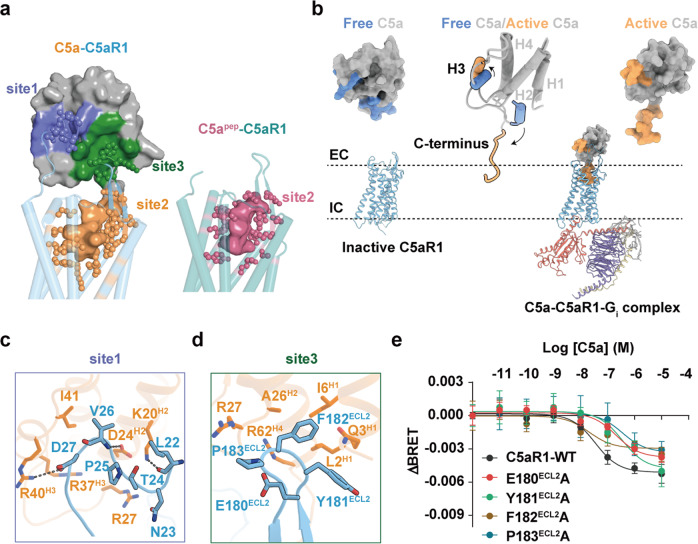
Three-site binding mode of C5a to C5aR1. **a** The recognition of C5aR1 by C5a with a three-site binding mode (left panel), and by C5a^pep^ with a one-site binding mode (right panel). The agonists C5a and C5a^pep^ are shown as surface, and C5aR1 is shown as cartoon with cylindrical helices. The binding sites in C5aR1 are shown as sticks. **b** The H3 and C-terminus of C5a undergo conformational changes from free state (PDB: 1KJS) to active state. EC, extracellular side; IC, intracellular side. **c**, **d** The interactions between C5a and C5aR1 at site 1 (**c**) and site 3 (**d**). The detailed interactions are shown as sticks. The salt-bridge and hydrogen-bond interactions are shown as black dashed lines. Sandy brown, C5a; light sky blue, C5aR1. **e** The representative dose response curves of the C5a-induced BRET ratio in HEK293 cells overexpressing FlAsH-BRET S2 wild type and corresponding mutants. Values are the means ± SEM from three independent experiments performed in triplicate.

Unlike the two-site binding mode (sites 1 and 2) of C5a with C5aR1 predicted from previous mutagenesis studies,^18,22^ the cryo-EM structure of the C5a–C5aR1 signaling complex reveals a three-site binding mode (Fig. 2a). Compared to the apo structure of C5a,^23^ receptor-bound C5a exhibits substantial conformational changes: first, helix 3 (H3) spins in an antiparallel orientation with H2, resulting in the exposure of a positively charged H2–H4 cavity that induces extensive electrostatic interactions and van der Waals forces with the membrane-proximal N-terminal region of C5aR1 (termed “site 1”) (Fig. 2b, c; Supplementary information, Fig. S5a). Second, the C-terminus, which adopts a 1.5-turn helix in apo C5a, releases into the core of the helical bundle upon receptor binding (“site 2”; Fig. 2b) and assumes a binding pose similar to that of C5a^pep^ (Fig. 2a). Third, a novel binding site (“site 3”) where the C5aR1 extracellular loop (ECL) 2 region occupies the C5a cavity, which previously accommodated the apo-state C-terminal tail, and packs with H1 and H2 of C5a (Fig. 2a, d), thereby further stabilizing the activation of major complement components.

### Functional role of binding site 3

Structural inspection of the C5a–C5aR1 complex revealed that F182^ECL2^ at site 3 fills the hydrophobic cavity formed by L2^H1^, I6^H1^, and A26^H2^ of C5a (Fig. 2d), while Y181^ECL2^ and P183^ECL2^ engage in van der Waals interactions with R27^H4^ and Q3^H1^ of C5a (Fig. 2d). Similar to C5aR1, ECL2 regions of chemokine receptors also adopt a β-hairpin secondary structure and participate in chemokine recognition (Supplementary information, Fig. S5b).^24,25^ Since ligand binding to the 7-transmembrane (TM) bundle of class A receptors results in extracellular conformational changes,^26^ we investigated the role of site 3 in C5a binding by monitoring the conformation of ECL regions upon binding using a fluorescein arsenical hairpin-bioluminescence resonance energy transfer (FlAsH-BRET) assay (Supplementary information, Fig. S5c, d). We screened five insertion sites (S1–S5) of FlAsH motifs in specific positions of each ECL and found that C5a–C5aR1 binding led to separation of the ECLs from the N-terminus in a concentration-dependent manner (Supplementary information, Fig. S5e–g). Moreover, alanine substitutions at residues E180^ECL2^, Y181^ECL2^, F182^ECL2^, and P183^ECL2^ resulted in substantial impairment of extracellular conformational changes (Fig. 2e; Supplementary information, Figs. S5h, S6), suggesting that ECL2 contributes to C5a binding. Our findings agree with a previous mutagenesis study that identified ECL2 as an essential component in C5aR1 activation.^27^

Structural comparison of the binding sites on C5aR1 of C5a^pep^ with those of C5a revealed a relative lack of contacts with ECL2 site 3 (Fig. 2a). Previous studies suggested that the ECL2 extracellular residues in aminergic receptors (i.e., β2AR, DRD2, and 5-HT2B) participate in β-arrestin bias modulation;^28–30^ thus, we next explored the contribution of the ECL2 of C5aR1 to the functional selectivity of signaling. Using cell-based assays, we found that four consecutive alanine substitutions of E180^ECL2^ to P183^ECL2^ resulted in a significant bias toward G_i_ protein signaling and away from β-arrestin recruitment and internalization; single alanine mutations (E180^ECL2^A, Y181^ECL2^A, F182^ECL2^A, and P183^ECL2^A), meanwhile, resulted in discrepant outcomes (Supplementary information, Figs. S5i, S6). Together, the results of our mutagenesis and cell-based assays revealed that C5a–C5aR1 interactions at site 3 are likely to be involved in ligand binding and signal transduction.

### Molecular recognition of C5a and C5a^pep^ at C5aR1 site 2

**Fig. 3:**
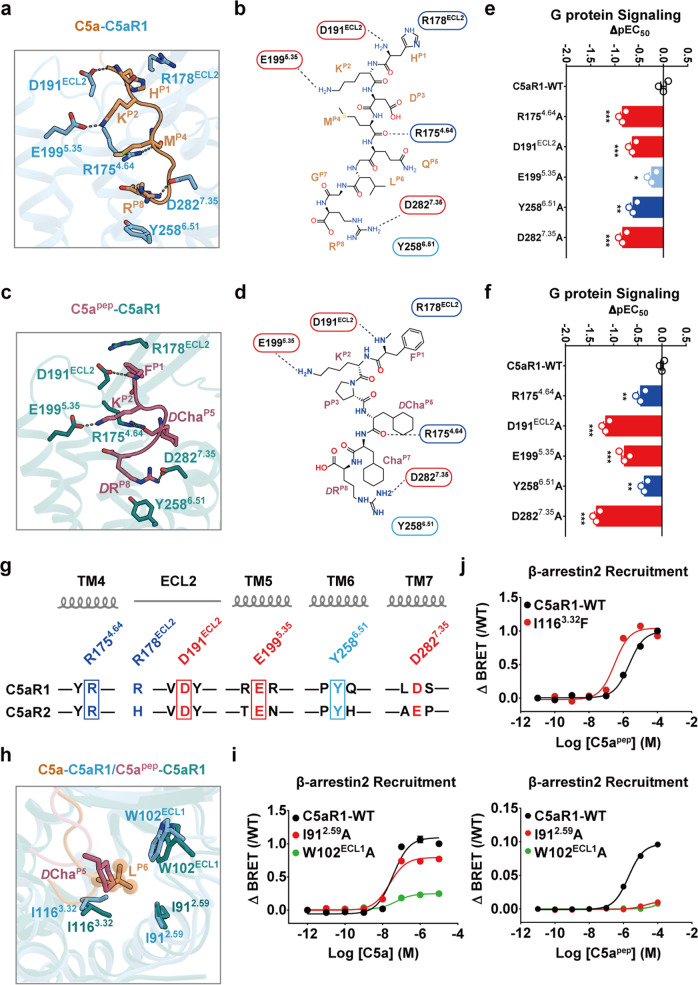
Common and specific interactions between C5a and C5a^pep^ in site 2. **a**–**d** Structural representation of common interaction sites between C5aR1 and C5a (**a**, **b**) or between C5aR1 and C5a^pep^ (**c**, **d**). The hook-shaped C-terminal tail of C5a or C5a^pep^ was anchored by polar or hydrophobic interactions with the residues R178^ECL2^, D191^ECL2^, E199^5.35^, R175^4.64^, D282^7.35^ and Y258^6.51^ in C5aR1. The detailed interactions between C5aR1 and C5a or C5a^pep^ are shown as 3D sticks (**a**, **c**) and 2D diagrams (**b**, **d**), respectively. Polar interactions are highlighted as black dashed lines. **e**, **f** The effects of mutations of common interaction sites on C5a (**e**) and C5a^pep^ (**f**) induced G_i_ protein signaling of C5aR1 examined by cAMP inhibition assay. Bars represent differences in calculated potency (ΔpEC_50_) for each mutant shown as percentage of the maximum in wild type. Data are the means ± SEM from at least three independent experiments, performed in triplicate and analyzed using one-way analysis of variance with Dunnett’s multiple comparison test to determine significance (compared with wild type). **P < 0.01, ***P < 0.001. **g** Sequence alignment of C5aR1 and C5aR2. The conserved common interaction sites are highlighted with squares. Residue positions labeled by Ballesteros**–**Weinstein numbering in C5aR1 are shown at the top. **h** Structural alignment of C5a-bound and C5a^pep^-bound C5aR1 showing that L^p6^ inserts into a hydrophobic cleft formed by I91^2.59^, W102^ECL1^ and I116^3.32^. Instead, Cha^P5^ of C5a^pep^ cannot stretch out to the corresponding position of L^P6^ in C5a, leading to weaker interactions. **i** The I91^2.59^A and W102^ECL1^A mutations greatly impaired C5a^pep^-induced β-arrestin 2 recruitment compared with that of C5a; the signaling was monitored by BRET assay. Data are the means ± SEM from at least three independent experiments performed in triplicate. **j** The I116^3.32^F mutation increased the C5a^pep^-induced β-arrestin 2 recruitment detected by BRET assay. Data are the means ± SEM from at least three independent experiments performed in triplicate.

As revealed by our structural comparison, the C-terminal regions of both C5a and C5a^pep^ fold into a hook shape at site 2 and are anchored by a set of almost identical interactions involving ECL2 and TM4–TM7 (Fig. 3a–d; Supplementary information, Fig. S7a–f), enabling the ligands to penetrate deeply into the TM helical bundle of C5aR1. Specifically, the main chains of H^P1^ in C5a and F^P1^ in C5a^pep^ form hydrogen bonds with the D191^ECL2^ side chain in C5aR1, with their side chains pointing from the P1 position toward the ECL2 region (Fig. 3a–d). The hooked C-termini are further stabilized by intramolecular salt bridges between K^P2^ and E199^5.35^ and hydrogen bonds between the guanidinium group of R175^4.64^ and the main chain of either M^P4^ in C5a or *D*Cha^P5^ in C5a^pep^ (Fig. 3a–d). The R^P8^ residue was particularly highly conserved, appearing in most peptide ligands of C5aR1 (Fig. 1a), and provided substantial binding affinity.^31^ Additionally, the R^P8^ side chain projects over the aromatic side chain of Y258^6.51^ into TM6 and TM7, forming a hydrophobic cation–π interaction (Fig. 3a, c). A critical hydrogen bond was identified between R^P8^ and D282^7.35^: the replacement of the latter with alanine significantly impaired the efficacy of C5a- and C5a^pep^-mediated activation (Fig. 3e, f; Supplementary information, Fig. S7g and Table S2). In agreement with this observation, C5aR1 has been demonstrated to exhibit compromised affinity for C5a-des-R, a naturally occurring C5a variant that lacks the C-terminal arginine residue (Fig. 1a).^31,32^

We next found that mutating the main residues of C5aR1 to alanines (i.e., R175^4.64^A, D191^ECL2^A, E199^5.35^A, Y258^6.51^A, and D282^7.35^A) reduced its potency upon C5a or C5a^pep^ stimulation by 3–50-fold (Fig. 3e, f; Supplementary information, Fig. S7g and Table S2), which is consistent with the common recognition of C5a and C5a^pep^. Moreover, the FlAsH-BRET assay results revealed that the extracellular conformational changes were significantly impaired by alanine substitutions of the common anchor residues (Supplementary information, Fig. S7h), which indicates the contribution of the anchor residues to C5a binding. Meanwhile, sequence alignment revealed that C5aR2 site 2 residues involved in ligand anchoring are highly conserved (Fig. 3g). The contribution of these anchors to downstream signaling was demonstrated in our mutagenesis experiments and functional assays, with replacement of the corresponding key C5aR2 anchor residues impairing the potency and efficacy of β-arrestin recruitment (Supplementary information, Fig. S7i and Table S3). Together, the combination of these structural and biochemical analyses suggest a similar ligand-binding mode between C5aR1 and C5aR2.

There were also some differences in the C-terminal conformations of C5a and C5a^pep^ that were observed. R^P8^ in C5a^pep^ occupies the bottom of the orthosteric site, connecting TM4 and TM7 of C5aR1 by forming hydrogen bonds with the S171^4.60^ side chain and salt bridges with D282^7.35^; this leads to subtle inward displacement of TM4 and TM7 and a smaller volume of site 2 compared with that in the C5a-bound receptor (Supplementary information, Fig. S7j). In agreement with our structural investigation, the S171^4.60^A mutation significantly impaired C5a^pep^-induced cAMP inhibition but had no effect on the response to C5a (Supplementary information, Fig. S7k and Table S2). Additionally, K279^7.32^ forms an ionic bond with the main chain of P^P3^ in C5a^pep^ instead of the side chain of D^P3^ in C5a (Supplementary information, Fig. S7l). In particular, L^P6^ in C5a binds tightly on the other side of site 2 to a hydrophobic cleft between TM2 and TM3, which is composed of I91^2.59^, W102^ECL1^, and I116^3.32^ in C5aR1 (Fig. 3h); conversely, such interactions in this region were not very strong during C5a^pep^ recognition due to the lack of a protrusion similar to L^P6^ in C5a in the contracted pose of the *D*Cha^P5^ side chain (Fig. 3h). Substantial conformational displacement of the side chains of W102^ECL1^ and I116^3.32^ was observed in the structures of C5a- or C5a^pep^-bound C5aR1 (Fig. 3h). When we increased the distance between residue^P6^ in the ligand and W102^ECL1^ or I91^2.59^ in C5aR1 by alanine replacement, C5a^pep^-induced β-arrestin recruitment was significantly impaired compared to that stimulated by C5a (Fig. 3i; Supplementary information, Tables S3, S4). C5a^pep^ has been shown to be a partial agonist of β-arrestin recruitment, relative to C5a.^17^ In our study, we found that the potency of β-arrestin recruitment increased when the isoleucine residue at position 3.32 in C5aR1 was mutated to a bulkier phenylalanine residue (Fig. 3j; Supplementary information, Table S4). Together, our results support the hypothesis that the distance between the side chain of position 3.32 in C5aR1 and position P6 in the ligand determines the degree of bias toward β-arrestin signaling.

### Ligand-binding and functional bias of C5aR1

**Fig. 4:**
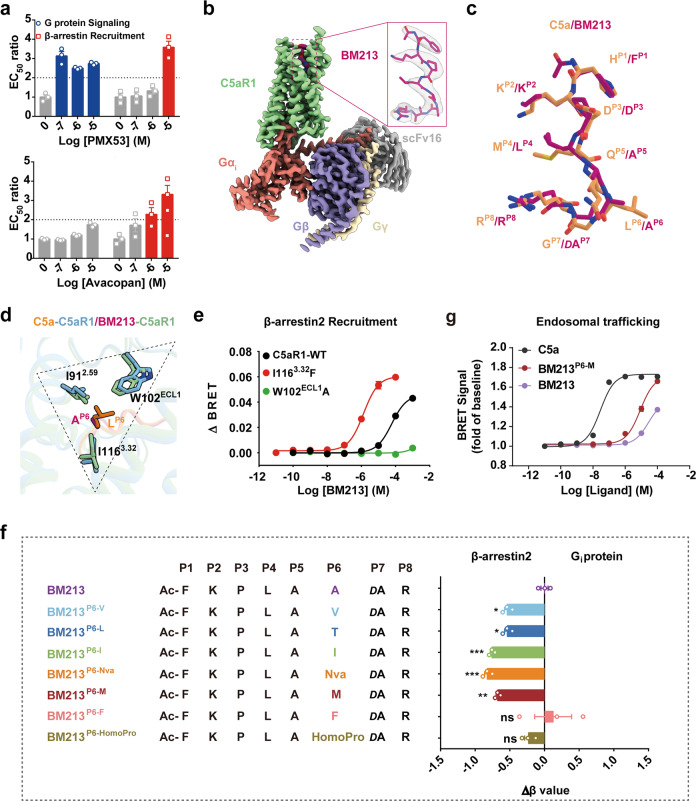
Structural basis of the biased agonism induced by BM213. **a** The biased antagonism induced by orthosteric ligand PMX53 (upper panel) and allosteric ligand Avacopan (lower panel). The EC_50_ values are derived from curve fit parameters from Supplementary information, Fig. S8b, c. Data are presented as the means ± SEM of three independent experiments performed in triplicate. **b** Cryo-EM maps of BM213-bound C5aR1**–**G_i_ complexes. Medium violet red, BM213; pale green, BM213-bound C5aR1; salmon, Gα_i_; slate blue, Gβ; wheat, Gγ; dark gray, scFv16. **c** Structural alignment of BM213 with C5a. The superimposition was based on the receptor. Sandy brown, C5a; hot pink, BM213. **d** Structural comparison of the interactions between L^P6^ of C5a and C5aR1 with those between A^P6^ of BM213 and C5aR1. **e** Representative curves for effects of the I116^3.32^F and W102^ECL1^A mutations in C5aR1 on BM213-induced β-arrestin 2 recruitment detected by BRET assay. Data are presented as the means ± SEM of three independent experiments performed in triplicate. **f** Substitution of A^P6^ in BM213 with bulkier residues induces β-arrestin 2-biased signaling relative to BM213. Bias factors were derived from curve fit parameters from Supplementary information, Fig. S10a, and were calculated using the endogenous agonist C5a as the reference. Statistical differences were determined by one-way ANOVA followed by the Dunnett’s multiple comparison test compared with BM213. *P < 0.05 **P < 0.01, ***P < 0.001; ns, no significance. Data are presented as the means ± SEM of three independent experiments performed in triplicate. **g** BRET-based agonist-dependent endosomal trafficking analysis of C5aR1 in HEK293 cells transfected with FYVE-mVenues. BRET ratio (30 min) is shown as fold of baseline; and data are the means ± SEM of three independent experiments performed in triplicate.

Since activated C5aR1 signals via G protein- and β-arrestin-dependent pathways, the therapeutic effects on both signaling pathways should be considered in drug design. The small-molecule antagonist avacopan, which binds to the extrahelical site of the receptor as an allosteric modulator, was recently approved by the U.S. Food and Drug Administration for the treatment of anti-neutrophil cytoplasmic autoantibody-associated vasculitis.^33^ Meanwhile, despite reaching adequate serum levels in clinical trials, no orthosteric antagonist (e.g., PMX53) has produced improved clinical outcomes, possibly because of on-target side effects.^34,35^ Here, we determined pharmacological characteristics of the antagonism: the results showed that avacopan exhibited biased inhibition toward β-arrestin recruitment over G protein coupling, whereas the opposite was observed for PMX53 (Fig. 4a; Supplementary information, Fig. S8a–c). Thus, deciphering the biased ligand recognition by C5aR1 is required to better understand the underlying mechanism.

Octapeptide BM213 was recently demonstrated to be a G protein-biased agonist of C5aR1 and shares a high sequence similarity with the C-terminus of C5a^36^ (Fig. 1a). Additionally, the loss of β-arrestin 2 recruitment in a previous study was shown to be due to L^P6^A substitution in BM213.^36^ Our results indicated that the distance between position P6 in the agonist and the TM2–ECL1–TM3 or IWI region in C5aR1 contributes to receptor-biased signaling. Therefore, to investigate the biased mechanism by which C5aR1 senses BM213, an additional cryo-EM structure of the BM213-bound C5aR1–G_i_ protein complex was solved at a 2.9 Å resolution (Fig. 4b; Supplementary information, Fig. S9).

BM213 resembles a hook shape and engages similar anchors to C5a in C5aR1 site 2 (Fig. 4c). The A^P6^ residue in BM213, which has a smaller side chain, shares the direction of L^P6^ in C5a and projects into the same hydrophobic pocket (Fig. 4d). Our β-arrestin recruitment assay results revealed that the I116^3.32^F mutation significantly increased and the W102^ECL1^A mutation strongly impaired the efficacy of β-arrestin recruitment (Fig. 4e; Supplementary information, Table S4). Taken together, our structural observations and functional assays further indicated that the ligand^P6^–C5aR^IWI^ pattern was involved in the biased mechanism of C5aR1, facilitating the precise molecular design and application of biased therapeutics for C5aR1.

Next, based on the structure and signaling profile of the C5aR1–BM213 complex, we next introduced specific mutations at position P6 and measured their effects on G_i_ protein activation and β-arrestin recruitment (Fig. 4f; Supplementary information, Fig. S10a), with the mutated peptides expected to interact with the C5aR1^IWI^ region. The BM213^P6-V^, BM213^P6-L^, BM213^P6-I^, BM213^P6-Nva^, and BM213^P6-M^ peptides all exhibited increased β-arrestin-biased features compared to BM213 (Fig. 4f; Supplementary information, Fig. S10a and Table S5), indicating that hydrophobic residues present at P6 may be required for effective β-arrestin recruitment. This finding is consistent with a previous study in which increased length/bulkiness of the side chain at this position removed the signaling bias.^36^ We also replaced smaller aliphatic residues with phenylalanine or pipecolic acid, and BM213^P6-F^ and BM213^P6-HomoPro^ demonstrated the same signaling profile as BM213 (Fig. 4f; Supplementary information, Fig. S10a and Table S5). One possible explanation is that the bulkier side chain at P6 may disrupt contact with critical residues in the C5aR1^IWI^ region.

Using quantitative analysis of endosomal trafficking, we then investigated the different β-arrestin 2 trafficking patterns induced by ligands of varying β-arrestin bias. Results of the bystander bioluminescence resonance energy transfer (BRET) assay revealed that the C5aR1 internalization increased in a concentration-dependent manner when stimulated with the different ligands, with BM213^P6-M^ eliciting higher internalization efficacy compared to that of BM213 (Fig. 4g). Confocal microscopy of mCherry-tagged β-arrestin 2 showed that C5a induced the localization of β-arrestin 2 to the plasma membrane at early time points when no significant BM213-induced trafficking of β-arrestin 2 was present. However, at subsequent time points, substantial internalization of β-arrestin 2 was induced by C5a while BM213 induced limited cell-surface recruitment. Consistent with our pharmacological assay results, we observed that BM213^P6-M^ stimulation induced faster β-arrestin 2 trafficking than BM213, indicating that β-arrestin 2 was first localized to the plasma membrane and then to endosomal vesicles (Supplementary information, Fig. S10b). Taken together, we characterized the profile of the G protein-biased ligand BM213 and demonstrated that the ligand^P6^–C5aR1^IWI^ pattern predicted biased signaling of the receptor, facilitating the design of biased ligands as potential therapeutics.

### Structural basis of C5aR1 activation

**Fig. 5:**
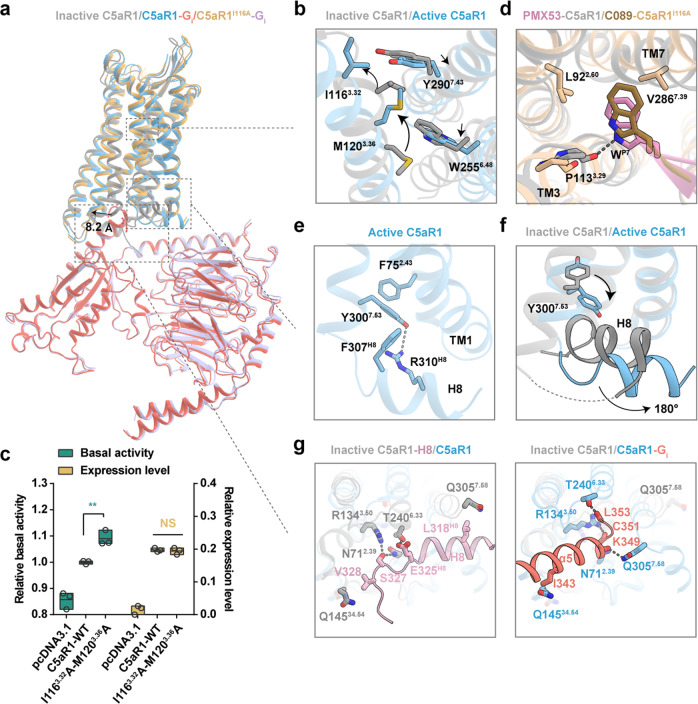
The activation mechanism of C5aR1. **a** Structural superposition of C5aR1**–**G_i_ complex (C5a-bound), C5aR1^I116A^**–**G_i_ complex (C089-bound) and inactive C5aR1 (PDB: 6C1R). **b** Structural comparison of the orthosteric binding pocket of inactive C5aR1 and active C5aR1. In particular, side chains of the residues I116^3.32^, M120^3.36^, W255^6.48^ and Y290^7.43^ are observed to exhibit a notable displacement in a “zipper-like” manner upon activation. The conformational changes of residues are shown as arrows. **c** The I116^3.32^A-M120^3.36^A mutation increases the basal activity of C5aR1. Statistical differences were determined by one-way ANOVA followed by the Dunnett’s multiple comparison test compared with C5aR1-WT. **P < 0.01; ns, no significance. Data are presented as the means ± SEM of three independent experiments performed in triplicate. **d** Structural alignment of PMX53**–**C5aR1 and C089**–**C5aR1^I116A^ shows that W^P7^ in C089 adopts a similar pose to that in PMX53, inserting into the cleft between TM3 and TM7, contacting with L92^2.60^, P113^3.29^ and V286^7.39^. **e** The non-canonical-state Y^7.53^ in active C5aR1 points toward TM1, and forms a hydrogen-bond with R310^H8^, as well as hydrophobic interactions with F75^2.43^ and F307^H8^. **f** Conformational comparison of Y^7.53^ and H8 between inactive C5aR1 and active C5aR1. H8 swings away from the center of cytoplasmic surface with ~180° of flipping upon activation. **g** Key residues involved in stabilizing the H8 helix in the inactive state (PDB: 5O9H) (left panel) and participating in binding α5 helix of G_i_ protein in the active state (right panel).

To understand the mechanism of complement cascade initiation, we structurally compared the activated and inactive C5aR1 (Fig. 5a).^37,38^ Substantial displacements were observed in the extracellular and intracellular tips of TM domains (Supplementary information, Fig. S11a), especially in the outward movement of TM6 in activated C5aR1, which is a hallmark of GPCR activation.^20,21^ Another feature of C5a and C5a^pep^ engagement with C5aR1 is the substantial rearrangement of TM3 and TM7 (Supplementary information, Fig. S11b), which results in an increased orthosteric binding volume in the activated receptor. A comparison of the distance between the upper half of TM3 and TM7 in GPCRs both supported the observed evidence (Supplementary information, Fig. S11c) and revealed a distinct feature of C5aR1 activation.

Although C5aR1 agonists and antagonists share similar binding modes at site 2 of the receptor, activation is selectively induced by the agonists. One explanation for this is that engagement of the L^P6^ side chain in C5a to P113^3.29^ in TM3 disrupts the direct ionic bond between the indole group in W^P7^ of the antagonist PMX53 (Supplementary information, Fig. S11d), which leads to upward rotation of TM3 upon receptor activation. Compared to the TM3s in the β2 adrenergic receptor and μ-opioid receptor, the conformational change of TM3 in C5aR1 demonstrates substantial rearrangement (Supplementary information, Fig. S11e), suggesting that it exhibits dynamic responses to different ligands. This rearrangement initiates substantial movement of two critical residues, I116^3.32^ and M120^3.36^, which tether the inactive configuration of TM3–TM6–TM7 by a set of hydrophobic stacking interactions with Y290^7.43^ and W255^6.48^, respectively, in a zipper-like manner. Repositioning of these residues results in the dissociation of the zipper, thereby allowing downward movement of W255^6.48^ and consequent outward movement of the intracellular end of TM6 (Fig. 5b). The I116^3.32^A-M120^3.36^A dual mutant significantly increased the basal activity of C5aR1 by disrupting intramolecular interactions within the zipper interface, indicating that the synergism of hydrophobic stacking dominates the inactive state of C5aR1 (Fig. 5c). Accordingly, weakening of the agonist-induced zipper lock initiates a cascade of conformational changes that magnify extracellular stimuli toward cytoplasmic effectors. In particular, the P-I-F motif (F251^6.44^ and I124^3.40^) is rearranged in agonist-bound C5aR1 structures, facilitating an outward swing of TM6 by 8.2 Å and thereby creating a cytoplasmic cavity for G protein coupling (Fig. 5a). Additionally, substantial displacement of position 3.40 upon activation was found in C5aR1 compared to the classic receptors (Supplementary information, Fig. S11f).

N292^7.45^ and N296^7.49^ in TM7 coordinate with a sodium ion with D82^2.50^ of TM2, stabilizing the inactive conformation of C5aR1 (Supplementary information, Fig. S11g).^38^ The resulting polar network, which is composed of sodium and surrounding residues, is disrupted during receptor activation, leading to substantial inward displacement of N296^7.49^ toward the helical bundle and shortening of the distance between N296^7.49^ and L127^3.43^ at the cytoplasmic ends of TM7 and TM3, respectively (Supplementary information, Fig. S11h).

### Mechanical activation switch in C5aR1

A previous biochemical study demonstrated that the I116^3.32^ residue in the zipper motif can convert an antagonist into an agonist;^39^ for example, the linear hexapeptide antagonist C089, which is identical to C5a^pep^ except for an alanine-to-tryptophan replacement at the P7 position in C089 (Fig. 1a),^12^ can act as an agonist for the I116^3.32^A mutant (Supplementary information, Fig. S12a–c). We speculated that the A116^3.32^ mutant might create a sub-pocket to adopt the bulky indole group of C089, which then dissociates the hydrophobic core and triggers receptor activation. To investigate this, we first determined the cryo-EM structure of C089-bound C5aR1 (I116^3.32^A) in complex with the G_i_ protein at 2.8 Å resolution (Supplementary information, Fig. S13). The EM density of C089 revealed a binding pose identical to that of C5a^pep^ (Supplementary information, Fig. S14a). However, the side chains of the substituted W^P7^ and Cha^P5^ in C089 exhibit different poses, resulting in a subtle conformational change in the contact interface (Supplementary information, Fig. S14b); in particular, the W^P7^ side chain is in the TM3–TM7 cleft, directly contacting the side chains of L92^2.60^, P113^3.29^, and V286^7.39^ and generating a pose similar to that of the PMX53-bound inactive structure (Fig. 5d). Conversely, the ionic bond between the indole group and the main chain of P113^3.29^ is disrupted in the C089-bound structure (Fig. 5d), with the critical role of the A116^3.32^ mutation in dissociating the zipper interface and thereby facilitating the rearrangement of TM3 and TM7 that is required for activation. Overall, structural comparison of C089-bound C5aR1 (I116^3.32^A) with C5a^pep^-bound C5aR1 revealed a similar conformation of TM bundles in C5aR1 (Supplementary information, Fig. S14c), supporting the functional significance of the zipper interface as an activation switch of C5aR1.

To further investigate these findings, we prepared several derivatives of BM213 with modifications at the P7 position. As the original BM213 study reported, conversion of d-alanine to l-alanine at P7 substantially reduced downstream signaling,^36^ while another pharmacological analysis suggested increased aromaticity at this position attenuated C5aR1 activity.^39^ Our functional assays revealed that the BM213^P7-dI,^ BM213^P7-dL^, and BM213^P7-dF^ derivatives, which have bulkier side chains at P7, exhibited a moderate influence on the G_i_ protein signaling; meanwhile, C5aR1 stimulated with l-residues at the P7 position markedly impaired the activation efficacy (Supplementary information, Fig. S14d). Given that the side chains of l-residues at P7 project toward TM7, thereby probably losing any potential contact with the zipper interface from a predictive model, the impact of stereochemistry on receptor activity further supports the crucial role of the zipper interface as an activation switch of C5aR1 (Supplementary information, Fig. S14e).

### G_i_ protein-coupling site of C5aR1 reveals a noncanonical active conformation of intracellular TM7 and H8

The cytoplasmic tip of TM7 in certain class A GPCRs exhibits a conformational change upon agonist binding and substantial displacement of TM6.^40^ The consensus NPxxY motif participates in the formation of a cavity for G protein coupling. In particular, Y^7.53^, which extends toward the core of the TM bundles and interacts with residues 3.43, 3.46, and R^3.50^, strengthens the packing of TM3 with TM7.^41^ While the canonical conformation of Y^7.53^ is present in most activated GPCRs, a noncanonical state of Y300^7.53^ is observed in C5aR1–G_i_ protein signaling (Supplementary information, Fig. S15a) in which its side chain projects toward TM1 and forms a hydrophobic contact with F75^2.43^ (Fig. 5e). This conformation probably allows H8 to swing away from the center of the cytoplasmic surface with ~180° of flipping (Fig. 5f), facilitating the formation of unique interactions of Y300^7.53^ with both F307^H8^ and R310^H8^ in active C5aR1 (Fig. 5e). Along with H8 rearrangement, the N71^2.39^, R134^3.50^, Q145^34.54^, and T240^6.33^ residues, which directly interact with the unique reverse orientation of H8, participate in new contacts with α5 in G_i_ with an additional hydrogen bond established between Q305^7.58^ and K349 in α5 after G_i_ protein coupling to the receptor (Fig. 5g). Alanine replacement of these residues significantly impaired cAMP inhibition (Supplementary information, Fig. S15b and Table S2). H8 in the angiotensin type 2 receptor adopts a similar conformation by flipping over to interact with the cytoplasmic cavity,^42^ which may result in steric blockade of G proteins and β-arrestins from binding to the receptor (Supplementary information, Fig. S15c). Our structural comparison revealed that both the α5 helix of G_i_ protein and the unique conformation of H8 in the inactive state occupy the overlapping cytoplasmic cavity of C5aR1 (Supplementary information, Fig. S15d), with H8 returning to the classic membrane-bound conformation upon receptor activation.

To investigate the noncanonical conformation of TM7 and H8 observed in the cryo-EM structures, we performed 250-ns molecular dynamic (MD) simulations using C5a–C5aR1 with or without G_i_ protein as the starting model. The results showed that TM7 and the rotamer of Y^7.53^ were maintained in the original noncanonical orientation; however, differences were displayed in the conformational change of H8 in both simulations. The presence of G_i_ protein coupling contributed to the stabilization of H8, whereas notable conformational changes of H8 occurred in the absence of G_i_ protein (Supplementary information, Fig. S15e, f). Overall, visualization of the unusual conformation of Y300^7.53^ and the atypical features of H8 in C5aR1 facilitate the understanding of the molecular mechanism of the complement receptor signaling system.

## Discussion

In this study, we first characterized the landscape of the C5a–C5aR1 interaction using cryo-EM and revealed a three-site binding mode. The discovery of a novel third binding site expands on the traditional view of C5a recognition and provides a structural interpretation of the modulatory role of ECL2 in C5aR1.^27^ Moreover, we identified the ligand^P6^–C5aR1^IWI^ pattern, which contributed greatly to biased signaling of the receptor, and developed a series of peptide derivatives that exhibited β-arrestin-biased features to varying degrees. Extending these structural insights, we investigated the mechanism of C5aR1 activation, which is governed by interruption of the zipper interface and recruitment of Gi protein via the noncanonical active conformation of intracellular TM7 and H8. Collectively, these observations underscore the ligand binding and functional bias of C5aR1, identify the activation mechanism, and offer novel possibilities to explore the therapeutic potential of biased C5aR1 modulators in the future. Single or combined substitutions of the P6–P7 ligand side chains may provide novel drug candidates with functional selectivity.

Our structural observation of C5a^pep^, which has higher affinity than the other C5a fragments, elucidated how peptide modifications at a couple of residues impart higher affinity. Our structural inspection revealed that the side chains of *D*Cha^P5^ and Cha^P7^ in C5a^pep^ filled the hydrophobic side of the binding pocket, forming strong hydrophobic interactions with TM2 and TM3 (Supplementary information Fig. S16). However, some C5a fragment analogs (e.g., BM213) usually possess residues with smaller side chains at equivalent positions. This observation is consistent with a previous investigation of octapeptide C5a analogs in which higher binding affinity was achieved by replacement with hydrophobic amino acids at positions 4–6;^43^ furthermore, replacements at positions 4–6 with hydrophobic side chain-bearing Cha improved binding affinity, while incorporation of other bulkier hydrophobic residues (Phe or Tyr) decreased binding,^43^ suggesting that a possible clash could be induced by bulkier side chains at positions 4–6. Thus, converting octapeptides into hexapeptides avoids such steric hindrance while retaining a similar binding affinity. Finally, certain chirality at appropriate positions is also crucial for elevation of affinity. In the investigation of octapeptide C5a analogs, the l–l configuration of the Cha^P4^–Cha^P5^ analog possessed the highest binding affinity compared with other combinations (i.e., d–d, d–l, l–d).^43^ Some have also suggested that d-Ala replacement at the P7 position may slightly increase affinity.^36,43^ Future combinations of mutagenesis and functional data may provide more insights into the structure–activity relationship of these peptide analogs.

Another observation was that D282^7.35^ in C5aR1 is replaced with E273^7.35^ in C5aR2. The D282^7.35^E mutation in C5aR1 significantly diminished C5a-induced G_i_ protein signaling and was accompanied by a moderate influence on β-arrestin signaling (Supplementary information Fig. S17a and Tables S2, S3). As revealed by our structural inspection, D282^7.35^ in C5aR1 forms salt bridges with R74^P8^ and ionic bonds with Q71^P5^ of C5a (Supplementary information Fig. S17b). the longer side chain of E273^7.35^ in C5aR2 might provide steric hindrance in the binding of C5a, resulting in upper-region interactions that decrease the G protein recruitment ability of C5aR2. This finding may also explain why the affinity of C5a-des-R for C5aR2 is reported to be even higher than that of C5a, which is the opposite situation to that of C5aR1.^11^ However, this notion assumes that C5a adopts a similar conformation upon C5aR2 recognition, and further structural information of the complex is needed. Future structural determination of C5a-bound C5aR2 complex would provide more information to understand the differences between C5aR1 and C5aR2.

Taken together, the results of pharmacological assays and the recognition of C5a and C5a-derived peptides by C5aR1 provide insights into the diverse ligand-mediated biased signaling mechanisms of C5aR1 and may facilitate the design of novel therapeutic agents with functional bias or subtype selectivity.

## Materials and methods

### Ligand expression and purification

The procedures of expression and purification were performed as previously described.^44^ Briefly, the human *C5a* (hC5a) gene was cloned into vector pET-32a (Novagen) following the Trx sequence. His_6_-tag and a TEV protease cleavage site were inserted between Trx and hC5a. To avoid nonspecific disulfide cross-linking between C5a molecules during purification, the sole cysteine residue of hC5a that was not engaged in intramolecular disulfide bridges (Cys704; prepro-C5 numbering) was replaced by an arginine, a substitution that was often encountered in C5a from other species and which did not dramatically affect the biological activity of C5a towards hC5aRs.^45^ hC5a-L72Q was generated from the hC5a-pET-32a construct by site-directed mutagenesis.

All C5a constructs were transformed in Shuffle T7 Express *Escherichia coli* cells (Weidi Bio, EC2030S). The cells were grown at 37 °C in TB medium supplemented with ampicillin (100 μg/mL) and expression of the C5a proteins was induced by the addition of 0.5 mM IPTG followed by overnight incubation at 16 °C. The cells were harvested, resuspended in buffer A (50 mM HEPES, pH 7.5, 300 mM NaCl, 30 mM imidazole), and disrupted by sonication. After clarification by centrifugation, the supernatant was applied onto a 5 mL Ni column. After a high-salt wash (50 mM HEPES, pH 7.5, 1 M NaCl, 30 mM imidazole) to remove nonspecifically bound proteins, the Trx-His_6_-tagged proteins were eluted with buffer B (50 mM HEPES, pH 7.5, 300 mM NaCl, 500 mM imidazole, 1 mM PMSF). The Trx-His_6_ tag was subsequently removed by overnight incubation at 4 °C using a 1:25 (rTEV:protein) mass ratio of in-house prepared recombinant His_6_-rTEV during dialysis against buffer D (50 mM HEPES, pH 7.5, 300 mM NaCl, 0.5 mM EDTA). The cleaved proteins were separated from contaminants, His-rTEV and uncleaved C5a material were removed by a second Ni-column purification step. The protein samples were then concentrated and rediluted with 50 mM HEPES, pH 7.5 to give a final salt concentration of 100 mM NaCl. The samples were then applied onto a 5 mL Hitrap fast flow cation-exchange column (GE Healthcare Life Sciences). Elution was performed with a 100-mL linear gradient from 100 to 1000 mM NaCl. Pure samples were flash-frozen in liquid nitrogen and kept at –80 °C until use.

### Peptides

C5a^pep^, BM213, all BM213-derived peptides and C089 were chemically synthesized at Chinapeptides (http://www.chinapeptides.com) with purity above 95%.

### Constructs

The human C5aR1 (residues 1–330) or human C5aR1 mutant (residues 1–330, I116A) cDNAs were subcloned into the pFastBac1 vector with the haemagglutinin (HA) signal sequence followed by a Flag epitope tag at the N-terminus. Human Gα_i1_ with a MYC epitope tag at the N-terminus or human Gα_i1_ with four dominant-negative mutations (S47N, G203A, E245A, A326S)^46^ were cloned into the pFastBac1 vector. Human Gβ_1_ containing N-terminal hexahistidine-tag and human Gγ_2_ were cloned into a pFastBac-dual vector for co-expression. The synthetic cDNA of single-chain variable fragment scFv16 that was utilized to further enhance the nucleotide-free G_i_ complex stability was cloned into pFastBac1 vector containing a GP67 secretion signal before the N-terminus of the protein and hexahistidine-tag at the C-terminus.

### Expression and purification of scFv16

The procedures of expression and purification were performed as previously described.^21^ Briefly, the His-tagged scFv16 protein was expressed in the secreted form in *Spodoptera frugiperda (Sf9)* insect cells using the Bac-to-Bac Baculovirus system (Invitrogen) and purified by Ni-NTA resin. The eluted protein was further loaded into a size exclusion column (Superdex 200 16/60) equilibrated in a buffer consisting of 20 mM HEPES, pH 7.5, and 100 mM NaCl. The fractions containing pure protein were collected and concentrated at 10 mg/mL. Then the protein was flash-frozen in liquid nitrogen and was stored at –80 °C for further using.

### Expression and purification of G_i_ heterotrimer

The procedures of expression and purification of G protein heterotrimer were performed as previously described.^47^ Briefly, baculovirus encoding the Gα_i1_ with four dominant-negative mutations, and His-tagged Gβ_1_Gγ_2_ proteins were co-infected into *Sf9* cells at a density of 3.0 × 10^6^ cells/mL at a ratio of 2:1. Cells were collected 72 h after infection. The Gi heterotrimer was purified by Ni-NTA resin and the eluted protein was further loaded into a size exclusion column (Superdex 200 16/60) equilibrated in a buffer consisting of 20 mM HEPES, pH 7.5, 100 mM NaCl, 10% glycerol, 1 mM MgCl_2_, 1 µM GDP and 0.1 mM TCEP. Peak fractions containing pure protein were collected and concentrated at 2 mg/mL, then the protein was flash-frozen in liquid nitrogen and was stored at −80 °C for further use.

### Expression and purification of C5aR1–G_i_ complex

Baculovirus encoding the HA-FLAG-C5aR1, Gα_i1_, and Gβ_1_Gγ_2_ proteins were co-infected into *Sf9* cells at a density of 3.0 × 10^6^ cells/mL at a ratio of 1:2:1. Baculovirus encoding the Gα_i1_ with four dominant-negative mutations were co-infected with C5aR1^I116A^, and baculovirus encoding the Gα_i1_ with a MYC epitope tag at the N-terminus were applied in the rest of the cases. After 60 h, the cells were collected and lysed with 20 mM HEPES, pH 7.5, 50 mM NaCl, 5 mM MgCl_2_, 5 mM CaCl_2_, protease inhibitors (160 μg/mL benzamidine, 100 μg/mL leupeptin), and 10 μM ligands at room temperature for 2 h. The membrane fraction was then solubilized with 20 mM HEPES, pH 7.5, 100 mM NaCl, 5 mM MgCl_2_, 5 mM CaCl_2_, 0.5% lauryl maltose neopentyl glycol (LMNG; Anatrace) (w/v), 0.1% cholesteryl (w/v), 10% glycerin (w/v), protease inhibitors (160 μg/mL benzamidine, 100 μg/mL leupeptin), 10 µM ligands, 10 mg/mL scFv16, and 25 mU/mL Apyrase for 2 h at 4 °C. After high-speed centrifugation, the supernatant was subjected to affinity purification using M1 anti-FLAG antibody coupled to Sepharose beads. The resin was transferred to a gravity column and washed with 30 column volumes of the Flag resin wash buffer (20 mM HEPES, pH 7.5, 100 mM NaCl, 3 mM MgCl_2_, 5 mM CaCl_2_, 5 μM ligands, 0.01% (w/v) LMNG and 0.001% (w/v) cholesteryl hemisuccinate (CHS)). The bound complex was eluted with 10 column volumes of elute buffer containing 10 mM EDTA and 0.2 mg/mL FLAG peptide. The concentrated sample was loaded onto a Superose6 increase 10/300 size exclusion column or a Superdex 200 16/60 size exclusion column (GE Healthcare) that was pre-equilibrated in 20 mM HEPES, pH 7.5, 100 mM NaCl, 3 mM MgCl_2_, 0.00075% (w/v) LMNG, 0.00025% glyco-diosgenin (GDN; Anatrace) and 0.0001% (w/v) CHS, and 5 µM ligands. Peak fractions of the target complex were pooled and concentrated using an Amicon Ultra Centrifugal Filter (MWCO, 100 kDa).

### Cryo-EM grid preparation and data collection

Three microliters of the purified C5a–C5aR1–G_i_–scFv16, C5a^pep^–C5aR1–G_i_–scFv16, BM213–C5aR1–G_i_–scFv16, and C089–C5aR1^I116A^–G_i_–scFv16 at the concentration of 15 mg/mL, 20 mg/mL, 14 mg/mL and 25 mg/mL, respectively, were applied to glow-discharged 300 mesh Au grids (Quantifoil R1.2/1.3), subsequently blotted for 2–3 s with the paraments of 100% humidity at 4 °C and vitrified by flash-frozen in liquid ethane using a Vitrobot Mark IV (Thermo Fisher Scientific). Cryo-EM data collections of all four samples were performed on a Titan Krios electron microscope (Thermo Fisher Scientific) in the SKLB West China Cryo-EM Center, Sichuan University (Sichuan, China), at an accelerating voltage of 300 kV. Movies were recorded using a K2 Summit detector (Gatan) GIF equipped with a Quantum energy filter (operated with a slit width of 20 eV), with a nominal magnification of 165,000× in counting mode, resulting in a corresponding pixel size of 0.85 Å. The total exposure time was 6 s, and 30 frames were recorded per movie stack with a defocus range of –0.8 to –1.8 μm, leading to a total accumulated dose of 66, 62, 67, 63 e^–^/Å^2^ on C5a–C5aR1–G_i_–scFv16, C5a^pep^–C5aR1–G_i_–scFv16, BM213–C5aR1–G_i_–scFv16, and C089–C5aR1^I116A^–G_i_–scFv16, respectively. All the data was auto-collected by EPU software (Thermo Fisher Scientific), and a total of 4526, 4857, 5777, and 4255 movies were gained for C5a–C5aR1–G_i_–scFv16, C5a^pep^–C5aR1–G_i_–scFv16, BM213–C5aR1–G_i_–scFv16, and C089–C5aR1^I116A^–G_i_–scFv16, respectively.

### Image processing and 3D reconstruction

All the Cryo-EM image stacks were dose-weighted and aligned by MotionCor2,^48^ and contrast transfer function parameters for each micrograph were estimated by GCTF.^49^ The following data processing was performed by using RELION3.0^50^ and cryoSPARC 3.1.0.^51,52^

For the dataset of the C5a–C5aR1–G_i_–scFv16 complex, a total of 3,922,460 particles and 665,663 particles were auto-picked by Laplacian-of-Gaussian (LOG)-based auto-picking and template-based auto-picking, respectively. Subsequently, the particles were subjected to reference-free 2D classification for 2 rounds in RELION3.0; then well-defined particles were merged and the duplicates were removed. The remaining 3,074,759 particles were imported to cryoSPARC for ab initio reconstruction, and 1 round of heterogeneous refinement. The well-defined subsets were further processed by a round of 2D classification and heterogeneous refinement in cryoSPARC, followed by a round of 3D classification with mask on the complex except for the AHD of the Gα_i_, producing a good subset accounting for 623,262 particles. The selected particles were subsequently subjected to CTF refinement, Bayesian polishing, and 3D auto-refinement, yielding a map at 2.9 Å resolution according to the gold-standard Fourier shell correlation (FSC) using the 0.143 criterion. The final map was sharpened by DeepEMhancer.^52^ The local resolution was estimated in RELION3.0.

For the dataset of the C5a^pep^–C5aR1–G_i_–scFv16 complex, a total of 4,329,433 particles were auto-picked and subjected to 2D classification for 2 rounds in RELION3.0; the 2,184,568 well-defined particles were selected and imported to cryoSPARC for ab initio reconstruction and 1 round of heterogeneous refinement. After a further round of 3D classification, a selected subset containing 191,664 particles were processed by non-uniform refinement, yielding a final map at 3.2 Å resolution according to the 0.143 criterion of the FSC. The local resolution was estimated in cryoSPARC.

For the dataset of the BM213–C5aR1–G_i_–scFv16 complex, a total of 6,361,876 particles were auto-picked. After 2 rounds of 2D classification and 1 round of 3D classification, a good subset containing 2,314,659 particles was selected and processed by 3D auto-refinement. Further processing by CTF refinement, Bayesian polishing, and 3D auto-refinement, produced a 2.9-Å resolution map according to the 0.143 criterion of the FSC. The final map was sharpened by DeepEMhancer. The local resolution was estimated in RELION3.0.

For the dataset of C089–C5aR1^I116A^–G_i_–scFv16, a total of 2,955,107 particles were auto-picked by 2D reference-based auto-picking. After 1 round of 2D classification, the junky particles were discarded and the remaining particles were applied to a round of 3D classification with 3 classes. Particles from the best-resolved class were subjected to a further round of 3D classification focusing on the protein complex; two well-defined classes containing 298,883 particles were subjected to 3D refinement, Bayesian polishing, and CTF refinement, generating a 2.8-Å resolution map sharpened by DeepEMhancer. The local resolution was estimated in RELION3.0.

### Cryo-EM model building and refinement

The crystal structure of C5aR1 in the inactive state (PDB: 5O9H) was used to build the initial model of the C5aR1 receptor in C5a-bound C5aR1–Gi–scFv16.^37^ The atomic coordinates of heterotrimeric G_i_ and scFv16 from the cryo-EM structure of the S1P-bound S1PR3–Gi–scFv16 complex (PDB: 7EW3) were used as the initial template of the Gi–scFv16 complex.^53^ The initial model of C5a^pep^–C5aR1–G_i_–scFv16 and BM213–C5aR1–G_i_–scFv16 complexes were taken from the refined structure of C5a–C5aR1–G_i_–scFv16. Models of C5a–C5aR1–G_i_–scFv16, C5a^pep^–C5aR1–G_i_–scFv16, BM213–C5aR1–G_i_–scFv16, and C089–C5aR1^I116A^–G_i_–scFv16 complexes were docked into the corresponding cryo-EM maps by UCSF Chimera,^54^ followed by iterative rounds of adjustment in COOT^55^ and real-space refinement in Phenix program.^56^ The final model statistics were validated using MolProbity.^57^ Model overfitting was measured by refining a “shaken” model against one of the unfiltered half-maps and calculating the FSC of the refined models against the two half-maps. The refinement statistics were provided in Supplementary information, Table S1. Structural figures were prepared in UCSF Chimera, Chimera X,^58^ and PyMOL (https://pymol.org/2/).

### C5aR1–G_i_-mediated cAMP inhibition assay

The cDNA sequences of wild-type *C5aR1* were cloned into the expression vector pcDNA3.1(+) with a HA signal sequence followed by a Flag tag at the N-terminus. Mutants used in our study were generated by using the Q5 site-Directed Mutagenesis kit (NEB). All the constructs were verified by sanger sequencing. The constructs were transiently expressed in HEK293 cells using PEI transfection reagent (YEASEN, 40816ES02) according to the manufacturer’s instruction. Cells were harvested 48 h post transfection. Cell surface expression levels of receptors were measured by enzyme-linked immunosorbent assay (ELISA).

To measure cAMP inhibition effects on the forskolin-induced cAMP accumulation of C5aR1, the GloSensor cAMP assay (Promega) was performed as described in the previous study.^59^ Briefly, Flag-tagged wild-type human C5aR1 was co-expressed with GloSensor plasmid in HEK293 cells in 6-well culture plates. After at least 24 h, transfected cells were harvested and suspended in assay buffer Hank’s Balanced Salt Solution (HBSS) containing 10 mM HEPES, pH 7.4, with an additional 3% v/v dilution of the d-Luciferin-Potassium Salt (YEASEN, 40902ES01). The ligands used in this study were dissolved in H_2_O to a stock concentration of 10 mM and followed by serial dilution using HBSS solution immediately before ligand stimulation. After incubation at 37 °C for 2 h, cells were stimulated by ligand (diluted in assay buffer containing 2.5 μM forskolin) for 30 min at room temperature. For the antagonist activity, cells were incubated with C5a (10^–6^ to 10^–13^) with or without antagonist for 30 min at room temperature. Finally, the luminescence signals were counted on the Synergy H1 microplate reader (BioTek). Each measurement was repeated in at least three independent experiments, each in triplicate. Data were processed using the nonlinear regression (curve fit) dose-response function in GraphPad Prism 7 to calculate the values of *E*_max_ and EC_50_.

### BRET-based Gα_i1_–γ_2_ dissociation assay

The activation of C5aR1 induced by ligands was measured through BRET-based Gα_i1_ dissociation from Gβ_1_γ_2_ as previously described.^60^ HEK293 cells were plated in 6-well plates containing 600,000–800,000 cells per well and cultured 24 h at 37 °C in cell incubator. Cells were co-transfected with C5aR1 wild type, Gα_i1_-NanoLuc luciferase (Nluc), Gβ_1_ and Gγ_2_-mVenus plasmid at a ratio of 1:1:1:1 (500 ng each). After transfection for 24 h, cells were harvested and dispensed into 96-well white plates and allowed to culture overnight. One day later, growth medium was aspirated and replaced with 90 µL of BRET assay buffer (25 mM HEPES, 1 mM CaCl_2_, 140 mM NaCl, 2.7 mM KCl, 0.9 mM MgCl_2_, 0.37 mM NaH_2_PO_4_, 5.5 mM d-glucose, 12 mM NaHCO_3_, pH 7.0) containing 5 µM coelenterazine h (Nanolight, CAT# 301) for 60 min. The baseline BRET signal (460–485 nm and 520–545 nm) was detected by the Synergy H1 microplate reader (BioTek). Next, prepared ligands were added into cells for 10 min and the second BRET signal was measured. The BRET signal was calculated as the emission ratio of the acceptor (mVenus, 520–545 nm) to the donor (Nluc, 460–485 nm). Data were analyzed using a simulation dose-response in GraphPad Prism 7.

### BRET assay measuring β-arrestin 2 recruitment to C5a receptors

The C5a-mediated β-arrestin 2 recruitment to C5aR1 and C5aR2, respectively, was measured using BRET-based assay as previously described.^36,61,62^ Briefly, HEK293 cells were transiently transfected with C5aR1-Nluc and mVenus-β-arrestin 2 or C5aR2-mVenus and Nluc-β-arrestin 2 constructs using PEI transfection reagent (YEASEN, 40816ES02) according to the manufacturer’s instruction. At 24 h post transfection, the cells were gently detached and seeded (100,000/well) onto 96-well white plates in DMEM containing 5% FBS. On the next day, cells were washed once with Phosphate Buffered Saline (PBS) and incubated in HBSS containing 20 mM HEPES, pH 7.4, with coelenterazine h (5 μM, YEASEN, 40906ES02) for 1 h (37 °C, 5% CO_2_). The baseline BRET light emissions (460–485 nm and 520–545 nm) were monitored by the Synergy H1 microplate reader (BioTek). After the incubation of different diluted concentrations of agonist with or without antagonist for 10 min, the second BRET light emissions were measured and the ligand-induced BRET ratio was calculated by subtracting the acceptor (mVenus, 520–545 nm) over donor (Nluc, 460–485 nm) emission ratio of the vehicle-treated wells from that of the ligand-treated wells. We carried out the nonlinear regression (curve fit) dose-response function in GraphPad Prism 7 to yield the values of *E*_max_ and EC_50_.

### Bias calculation

The measurement of cAMP inhibition and β-arrestin recruitment in our study was performed as described. Besides, the parameters used below were based on the curve fits of the combined datasets described above. The bias factors (*β* value) were determined by applying the following equation:^63^

$${\beta}\,{{{{{{{\mathrm{value}}}}}}}} = \log \left( {\left[ {\frac{{E_{{{{{{{{\mathrm{max}}}}}}}}},{{{{{{{\mathrm{P}}}}}}}}1}}{{{{{{{{{\mathrm{EC}}}}}}}}_{50},{{{{{{{\mathrm{P}}}}}}}}1}}\frac{{{{{{{{{\mathrm{EC}}}}}}}}_{50},{{{{{{{\mathrm{P}}}}}}}}2}}{{E_{{{{{{{{\mathrm{max}}}}}}}}},{{{{{{{\mathrm{P}}}}}}}}2}}} \right]{{{{{{{\mathrm{ligand}}}}}}}} \times \left[ {\frac{{E_{{{{{{{{\mathrm{max}}}}}}}}},{{{{{{{\mathrm{P}}}}}}}}2}}{{{{{{{{{\mathrm{EC}}}}}}}}_{50},{{{{{{{\mathrm{P}}}}}}}}2}}\frac{{{{{{{{{\mathrm{EC}}}}}}}}_{50},{{{{{{{\mathrm{P}}}}}}}}1}}{{E_{{{{{{{{\mathrm{max}}}}}}}}},{{{{{{{\mathrm{P}}}}}}}}1}}} \right]{{{{{{{\mathrm{reference}}}}}}}}} \right)$$

where P1 is cAMP data and P2 is BRET data; *β* value > 0 means G_i_ protein-biased, and *β* value < 0 denotes *β*-arrestin-biased.

Statistical differences were determined by two-sided, one-way ANOVA with Dunnett’s multiple comparison test.

### NFAT-RE luciferase assay

According to previous publications, the G_i_ basal activity of C5aR1 and its mutants was assessed by a membrane-proximal cell-based assay in HEK293 cells expressing C5aR1 constructs together with a chimeric G_qi_ protein (Gα_Δ6qi4myr_) that recognizes G_i_-coupled GPCRs but elicits G_q_-dependent phospholipase C activation.^64^ We measured NFAT-RE luciferase intensity as a readout of this chimeric G_i_ signaling. In brief, HEK293 cells were transiently co-transfected with C5aR1 constructs, 500 ng G_qi_ and 1000 ng NFAT reporter plasmids in 6-well plates, incubated at 37 °C in a 5% CO_2_ atmosphere for 24 h. Then the cells were dispensed into 96-well plates and seeded overnight. On the following day, luciferase activities were determined using luciferase assay kits (Vazyme, Cat# DD1204-01). Finally, the luciferase intensity was read on the Synergy H1 microplate reader (BioTek) using the luciferase program. The experiments were performed at least three times in triplicates. Data were processed using GraphPad Prism 7.

### FlAsH BRET assay

HEK293 cells were transfected with Nluc-C5aR1-FlAsH using PEI transfection reagent (YEASEN, 40816ES02) in 6-well plates according to the previous literature.^65^ Twenty-four hours later, the transfected cells were resuspended in 96-well plates and seeded overnight. Then the cells were washed with Opti-MEM and incubated with 2.5 μM FlAsH-EDT2 from the TC-FlAsH II In-Cell Tetracysteine Tag Detection Kit (Thermo Scientific, T34561) at room temperature for 40 min. The FlAsH-labeled cells were then washed with 1× BAL buffer and incubated in the HBSS containing coelenterazine h (working concentration, 5 μM) for 15 min. The ligand C5a (final concentration of 10^–12^–10^–5^ M) was added before reading BRET signals in the Synergy H1 microplate reader (BioTek), which was equipped with 460–485 nm excitation and 520–545 nm emission filters. The BRET ratio was calculated by dividing the 520–545 nm emission by the 460–485 nm emission. Data were processed using the nonlinear regression dose-response curves in GraphPad Prism 7 to calculate the values of *E*_max_ and EC_50_. The assay was performed three times independently in triplicate.

### ELISA for the receptor cell-surface expression

To measure the cell surface expression of the C5a receptors, ELISA was performed as described in the previous study.^63^ Briefly, plasmids of wild-type and mutant C5a receptors were transiently transfected as described above. At 24 h post transfection, the cells were gently detached and seeded onto poly-lysine-coated 96-well plates and incubated overnight. The next day, the cells were washed twice using PBS and fixed with 4% (w/v) paraformaldehyde followed by twice washing with PBS. Then, the cells were blocked with 5% (w/v) BSA at room temperature for 1 h. Anti-Flag HRP-conjugated monoclonal antibody was added to plates and incubated for 1 h at room temperature. Plates were washed with PBS and HRP substrate 3,3′,5,5′-tetramethylbenzidine was added. The reaction was quenched by adding 2 M H_2_SO_4_ solution and absorbance was read at 450 nm on the Synergy H1 microplate reader (BioTek). Each measurement was repeated in at least four independent experiments performed in triplicate. Values were normalized to the wild-type receptor and graphed as percentages of wild type using GraphPad Prism 7.

### Confocal microscopy

To visualize the ligand-induced β-arrestin recruitment, confocal microscopy was used following the protocol described previously.^17^ Briefly, HEK293 cells were co-transfected with C5aR1-WT and mCherry-β-arrestin 2 plasmids in 1:3 ratio by PEI transfection reagent (YEASEN, 40816ES02). After 24 h transfection, 5 × 10^5^ cells were seeded on 0.1 mg/mL poly-d-lysine precoated coverslips in 12-well plates. After 24 h, the cells were serum-starved for 6 h and then stimulated with C5a (100 nM), BM213 (100 μM), and BM213^P6-M^ (100 μM), respectively, for selected time points. After stimulation, the cells were washed once with 1× PBS. The cells were fixed with 4% (w/v) paraformaldehyde for 20 min at room temperature. After washing with 1× PBS, cells on coverslips were mounted on slides with Antifade Mounting Medium with DAPI (Beyotime, P0131-25 ml). Fluorescence images of all samples were acquired using Leica Stellaris 5 confocal microscope. Images were processed post imaging in the LAS X software suite from Leica. All the experiments were repeated at least three times independently on different days.

### Bystander BRET-based C5aR1 internalization assay

To quantify C5aR1 transit into early endosomes and ligand-dependent internalization properties, we adapted a “bystander” BRET assay according to the protocol previously described.^66^ Briefly, The NLuc domain was fused to the C-terminal tail of the C5aR1. HEK293 cells were co-transfected with C5aR1-Nluc and FYVE-mVenus at the ratio of 1:4 in 6-well culture plates. At 24 h post transfection, the cells were dispensed into 96-well plates and seeded overnight. On the following day, cells were washed once with PBS and incubated in the HBSS containing 20 mM HEPES, pH 7.4, with coelenterazine h (working concentration, 5 μM) for 60 min. The baseline BRET signals were detected by the Synergy H1 microplate reader (BioTek), which was equipped with 460–485 nm excitation and 520–545 nm emission filters. The second BRET signal was measured after adding the different diluted concentrations of agonist. The BRET ratio was calculated by dividing the 520–545 nm emission by the 460–485 nm emission. BRET ratio (30 min) is shown as fold of baseline, and we carried out the nonlinear regression (curve fit) dose-response function in GraphPad Prism 7.

### MD simulations

#### Simulation setup

The initial models for MD simulation were taken from C5a–C5aR1–G_i_ complex with or without G_i_ heterotrimer removed. The orientation of models was decided by running PPM 2.0.^67^ CHARMMGUI^68^ was used for building MD simulation systems and embedding models into 1-palmitoyl-2-oleoyl-sn-glycero-3-phosphocholine lipid bilayers in a regular hexagonal prism box with a size of approximately 7.0 nm × 7.0 nm × 18.8 nm and a rectangular box with a size of approximately 7.5 nm × 7.5 nm × 13.0 nm. 0.15 M NaCl was added to increase ion concentration, and the final systems were solvated in CHARMM TIP3P water model; the system charge was neutralized by using ions to replace water molecules. The following Amber force fields were applied: *ff14SB* for protein, *lipid17* for lipid.^69^

#### Simulation protocols

Velocities of individual atoms in systems were assigned randomly and independently. After energy minimization with steepest descent algorithm, the systems were equilibrated in the NVT ensemble for 250 ps with a time step of 1 fs followed by performing equilibration in NPT ensemble at 310.15 K and 1 bar. LINCS algorithm was used for constraining all bonds involving hydrogen atoms. Protein and ligand were restrained during equilibration, and the force constants were gradually decreased from 1000 kJ·mol/nm^2^ to 0. Finally, production runs were performed at constant pressure (1 bar) and temperature (310.15 K) with a time step of 2 fs. Electrostatic interactions were calculated using the particle-mesh Ewald method, and the nonbonded interactions were cut off at 1 nm. GROMACS (v.2019.6) was used to perform MD simulations,^70^ and MD trajectories were analyzed using VMD.^71^

## Supplementary information

Supplementary information, Fig. S1

Supplementary information, Fig. S2

Supplementary information, Fig. S3

Supplementary information, Fig. S4

Supplementary information, Fig. S5

Supplementary information, Fig. S6

Supplementary information, Fig. S7

Supplementary information, Fig. S8

Supplementary information, Fig. S9

Supplementary information, Fig. S10

Supplementary information, Fig. S11

Supplementary information, Fig. S12

Supplementary information, Fig. S13

Supplementary information, Fig. S14

Supplementary information, Fig. S15

Supplementary information, Fig. S16

Supplementary information, Fig. S17

Supplementary information, Table S1

Supplementary information, Table S2

Supplementary information Table S3

Supplementary information, Table S4

Supplementary information, Table S5

## Data Availability

The cryo-EM density maps and atomic coordinates have been deposited in the Electron Microscopy Data Bank (EMDB) and Protein Data Bank (PDB) under accession numbers EMD-33633 and 7Y64 for C5a–C5aR1–G_i_ complex; EMD-32634 and 7Y65 for C5a^pep^–C5aR1–G_i_ complex; EMD-33635 and 7Y66 for BM213–C5aR1–G_i_ complex; EMD-33636 and 7Y67 for C089–C5aR1^I116A^–G_i_ complex. All other data are available on request to the corresponding authors.
