# Supplementary material for: Mechanism of activation and biased signaling in complement receptor C5aR1
Source: Cell Res. 2023 Feb 17;33(4):312–24. doi: 10.1038/s41422-023-00779-2 (PMC9937529; doi:10.1038/s41422-023-00779-2)
Supplement: Supplementary file 1 — Supplementary information, Fig. S1 [file 41422_2023_779_MOESM1_ESM.pdf]

Supplementary information, Fig. S1

a

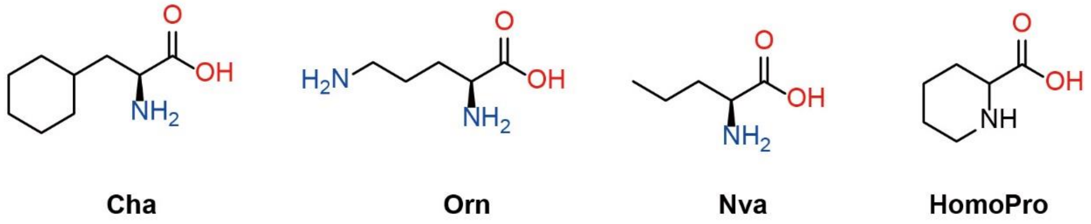

b

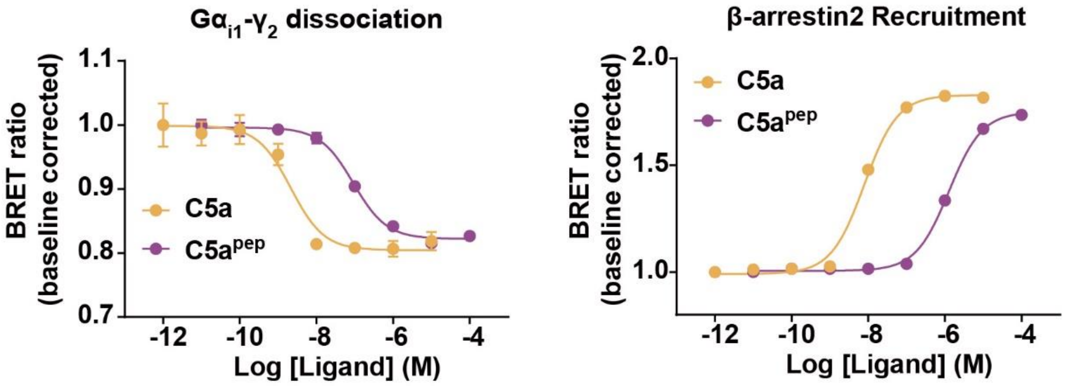

Fig. S1. C5a and C5a^{pep} induced G$_i$ and β-arrestin signaling.

a, The chemical structures of non-natural amino acids used in this study. Cha, Cyclohexylalanine; Orn, Ornithine; Nva, 2-Amino-3-methylbutyric acid; HomoPro, Homoproline.

b, C5a and C5a^{pep} induced G$_i$ and β-arrestin signaling. The G$_i$ protein signaling was detected by G$_i$ protein dissociation assays and the β-arrestin2 recruitment was examined by BRET assay. Data are presented as the mean ± SEM of three independent experiments performed in triplicate.
