# Supplementary material for: Mechanism of activation and biased signaling in complement receptor C5aR1
Source: Cell Res. 2023 Feb 17;33(4):312–24. doi: 10.1038/s41422-023-00779-2 (PMC9937529; doi:10.1038/s41422-023-00779-2)
Supplement: Supplementary file 4 — Supplementary information, Fig. S4 [file 41422_2023_779_MOESM4_ESM.pdf]

Supplementary information, Fig. S4

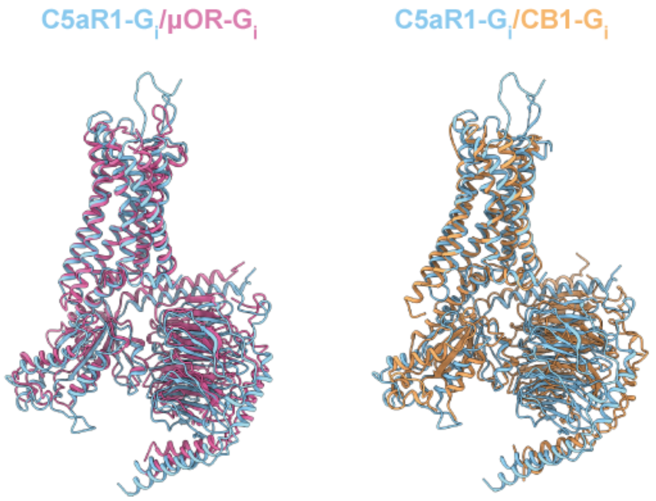

Fig. S4. Structural alignment of C5aR1-G$_i$ complex with μOR-G$_i$ complex and CB1-G$_i$ complex. The superimposition was based on the receptor. Light sky blue, C5aR1-G$_i$ complex; hot pink, μOR-G$_i$ complex (PDB:6DDE); orange, CB1-G$_i$ complex (PDB: 6N4B).
