# Supplementary material for: Mechanism of activation and biased signaling in complement receptor C5aR1
Source: Cell Res. 2023 Feb 17;33(4):312–24. doi: 10.1038/s41422-023-00779-2 (PMC9937529; doi:10.1038/s41422-023-00779-2)
Supplement: Supplementary file 5 — Supplementary information, Fig. S5 [file 41422_2023_779_MOESM5_ESM.pdf]

Supplementary information, Fig. S5

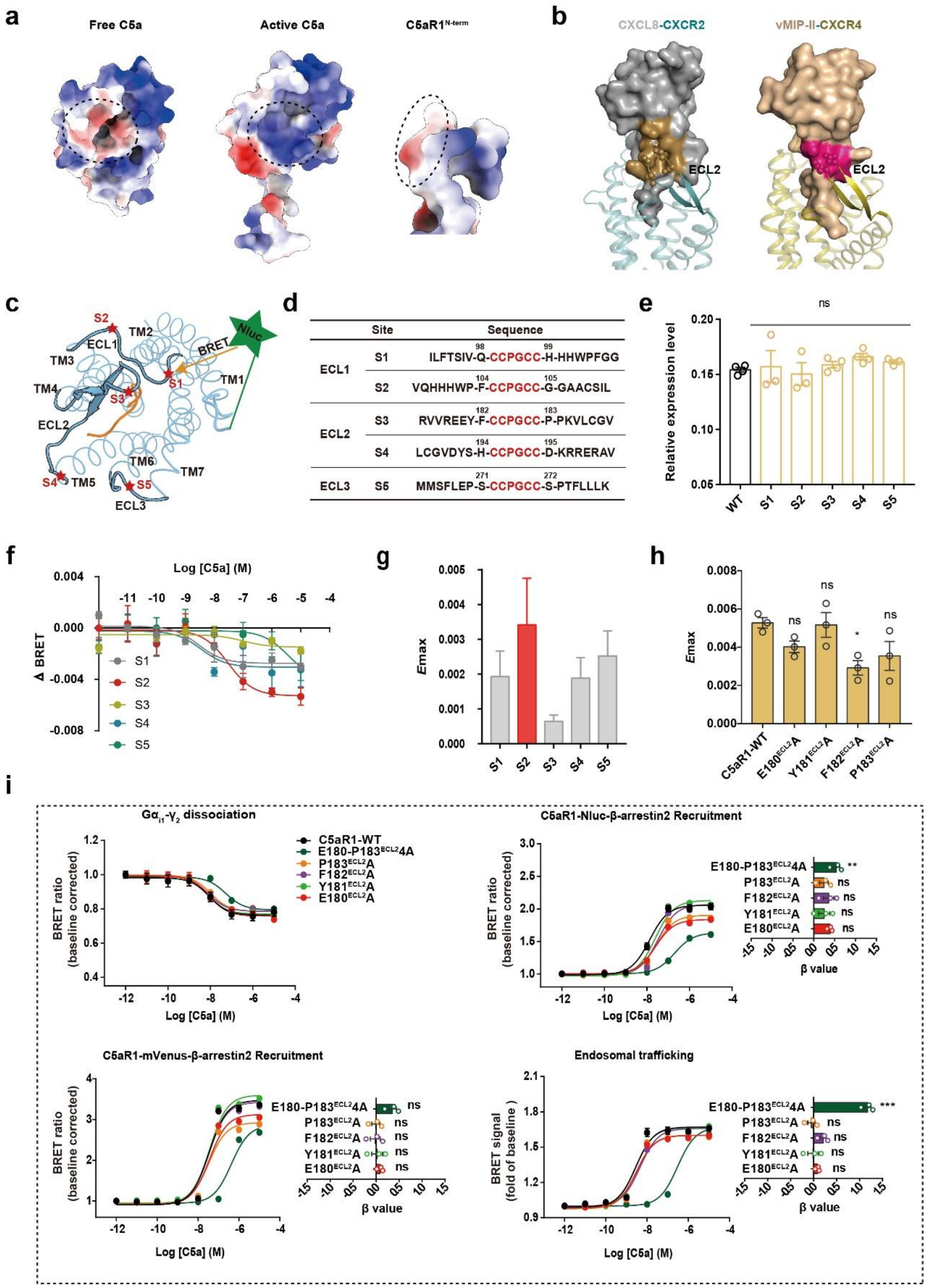

Fig. S5. Site 1 and Site 3 of C5a-C5aR1.

a, The electrical charge distribution of free state C5a (left panel), active state C5a (middle panel) and N-terminus of C5a-bound C5aR1 (right panel). The region of C5a interact with the N-terminus of C5aR1 were highlighted with black-dashed circles.
b, Both of ECL2 in CXCR2 (PDB: 6LFM) and CXCR4 (PDB:4RWS) involved in the ligand recognition, forming a binding site equivalent to the site 3 in C5a-bound C5aR1.
c, FlAsH-BRET assay design of C5aR1. The Nluc was fused to the N-terminus of C5aR1, and the FlAsH motif were inserted in 5 different extracellular loop regions. The designated positions were indicated as red stars.
d, The detail FlAsH motif incorporation sites in C5aR1. The FlAsH motifs are highlighted in red colors.
e, The expression levels of wild-type (WT) C5aR1 and five FlAsH motif incorporated FlAsH-BRET sensors detected by ELISA experiments. Data represent the mean ± SEM from three independent experiments performed in triplicate. ns, no significant difference.
f, The representative curve for C5a-induced BRET ratio in response to five C5aR1 FlAsH-BRET sensors. Values are mean ± SEM from three independent experiments performed in triplicate.
g, The efficacy changes for C5a-induced BRET ratio in response to five C5aR1 FlAsH-BRET sensors. Values are mean ± SEM from three independent experiments performed in triplicate.
h, The calculated efficacy (*E*max) changes upon C5a activation by different FlAsH-BRET S2 mutants.
i, The mutagenesis effects of residues in site 3 of C5aR1 on C5a induced different signaling pathways. The G_i protein signaling was examined by G_i protein dissociation assay, β-arrestin2 recruitment was detected by BRET assay including C5aR1 labeled with Nluc and mVenus and C5a-dependent endosomal trafficking of C5aR1 was measured by bBRET with FYVE-mVenus. The bias factors derived from curve fit parameters and show in the right side respectively. *P* values were determined by one-way ANOVA. The asterisk symbols indicate statistically significant difference (**P < 0.01, ***P < 0.001, ns: no significance). Data shown are mean ± SEM of a representative experiment performed in triplicate.
