# Supplementary material for: Mechanism of activation and biased signaling in complement receptor C5aR1
Source: Cell Res. 2023 Feb 17;33(4):312–24. doi: 10.1038/s41422-023-00779-2 (PMC9937529; doi:10.1038/s41422-023-00779-2)
Supplement: Supplementary file 6 — Supplementary information, Fig. S6 [file 41422_2023_779_MOESM6_ESM.pdf]

Supplementary information, Fig. S6

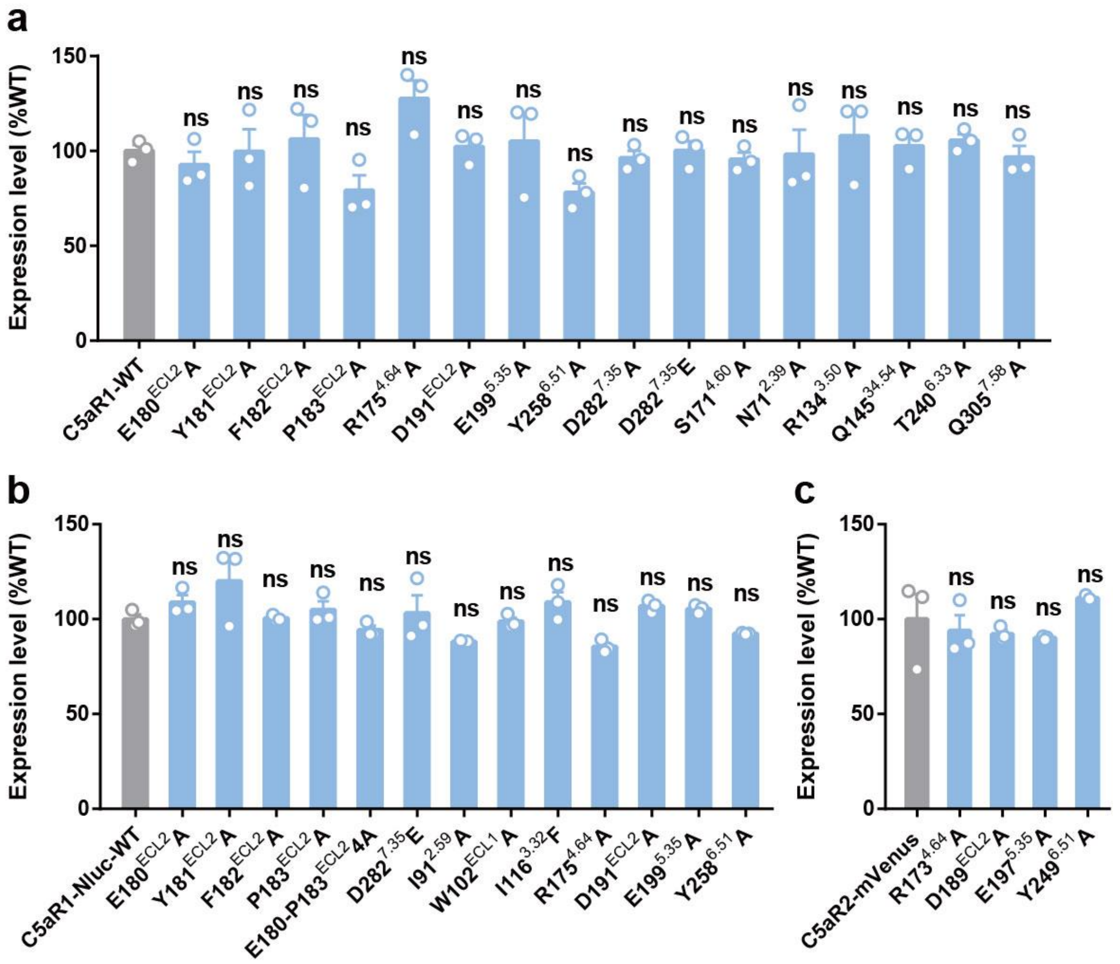

Fig. S6. The expression level of wild-type (WT) and mutants of C5aR1 and C5aR2. The cell surface expression level of the C5aR1(**a**), C5aR1-Nluc (**b**), and C5aR2-mVenus (**c**) WT and mutants were detected by ELISA assay. Data represent the mean ± SEM from three independent experiments performed in triplicate. ns, no significant difference.
