# Supplementary material for: Mechanism of activation and biased signaling in complement receptor C5aR1
Source: Cell Res. 2023 Feb 17;33(4):312–24. doi: 10.1038/s41422-023-00779-2 (PMC9937529; doi:10.1038/s41422-023-00779-2)
Supplement: Supplementary file 7 — Supplementary information, Fig. S7 [file 41422_2023_779_MOESM7_ESM.pdf]

Supplementary information, Fig. S7

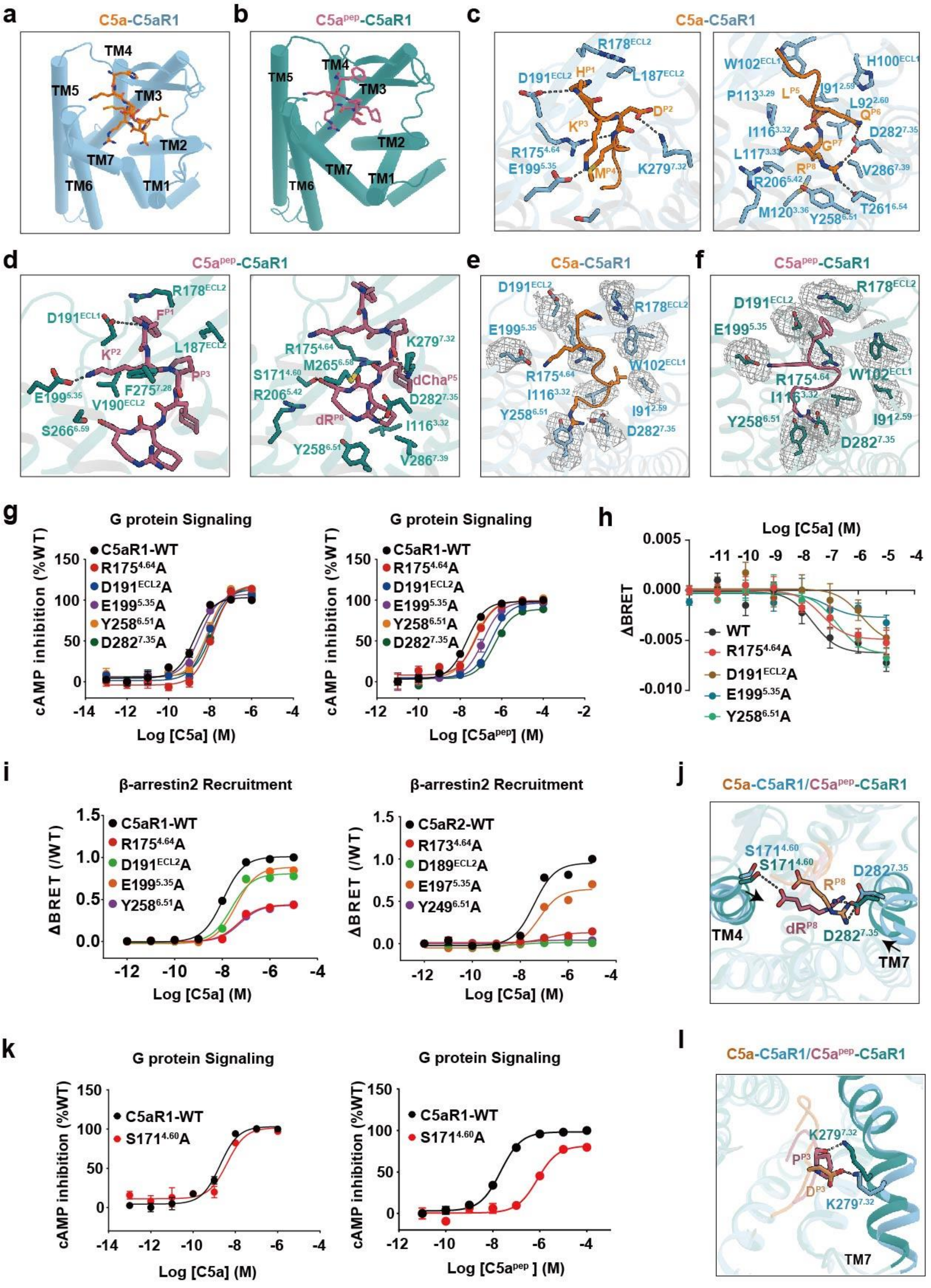

Fig. S7. Molecular details between C5aR1 and C5a or $C5a^{pep}$.

a-b, The C-terminus of C5a (**a**) and $C5a^{pep}$ (**b**) penetrate deeply into the TM helical bundle of C5aR1 in a similar binding pose. The C-terminus of C5a and $C5a^{pep}$ are shown as sticks, C5aR1 is shown as cartoon with cylindrical helices.

c-d, Detailed interaction of C5aR1 with C5a (**c**) or C5a^{pep} (**d**). The interactions between P1-P4 of C5a or C5a^{pep} and C5aR1 are shown in the left panel, that between P5-P8 of C5a or C5a^{pep} and C5aR1 are shown in the right panel. Polar interactions are highlighted as black dashed lines.

e-f, High-quality electron density facilitated unambiguous assignment of key residues in the binding pocket of C5a-bound (**e**) and C5a^{pep}-bound (**f**) C5aR1.

g, Representative curve for effects of the R175$^{4.64}$A, D191^{ECL2}A, E199$^{5.35}$A, Y258$^{6.51}$A and D282$^{7.35}$A mutations in C5aR1 on C5a (left panel) and C5a^{pep} (right panel) induced G protein signaling examined by cAMP inhibition assay. Data are presented as the mean ± SEM of three independent experiments performed in triplicate.

h, The representative dose response curves of the C5a-induced BRET ratio in HEK293 cells overexpressing FlAsH-BRET S2 wild type and corresponding mutants. Values are mean ± SEM from three independent experiments performed in triplicate.

i, Representative curve for effects of the R175$^{4.64}$A, D191^{ECL2}A, E199$^{5.35}$A and Y258$^{6.51}$A mutations in C5aR1 (left panel) and the R173$^{4.64}$A, D189^{ECL2}A, E197$^{5.35}$A and Y249$^{6.51}$A C5aR2 (right panel) on C5a induced β-arrestin2 recruitment examined by BRET assay. Data are presented as the mean ± SEM of three independent experiments performed in triplicate.

j, Structural alignment of C5a-bound and C5a^{pep}-bound C5aR1 showing that dR^{P8} in C5a^{pep} forms polar interactions with both S171$^{4.60}$ in TM4 and D282$^{7.35}$ in TM7, leading to inward shift of TM4 and TM7, but the R^{P8} in C5a lost the polar interactions with S171$^{4.60}$. Polar interactions are highlighted as black dashed lines.

k, The S171$^{4.60}$A mutation greatly impaired C5a^{pep}-induced G protein signaling (right panel) but had no effect on the potency and efficacy in response to C5a (left panel), the signaling was monitored by cAMP inhibition assay. Data are mean ± SEM from at least three independent experiments.

l, K279$^{7.32}$ of C5aR1 forms a salt-bride with the side chain of D^{P3} in C5a, instead a H-bond with the main chain of P^{P3} in C5a^{pep}. Polar interactions are highlighted as black dashed lines.
