# Supplementary material for: Mechanism of activation and biased signaling in complement receptor C5aR1
Source: Cell Res. 2023 Feb 17;33(4):312–24. doi: 10.1038/s41422-023-00779-2 (PMC9937529; doi:10.1038/s41422-023-00779-2)
Supplement: Supplementary file 8 — Supplementary information, Fig. S8 [file 41422_2023_779_MOESM8_ESM.pdf]

Supplementary information, Fig. S8

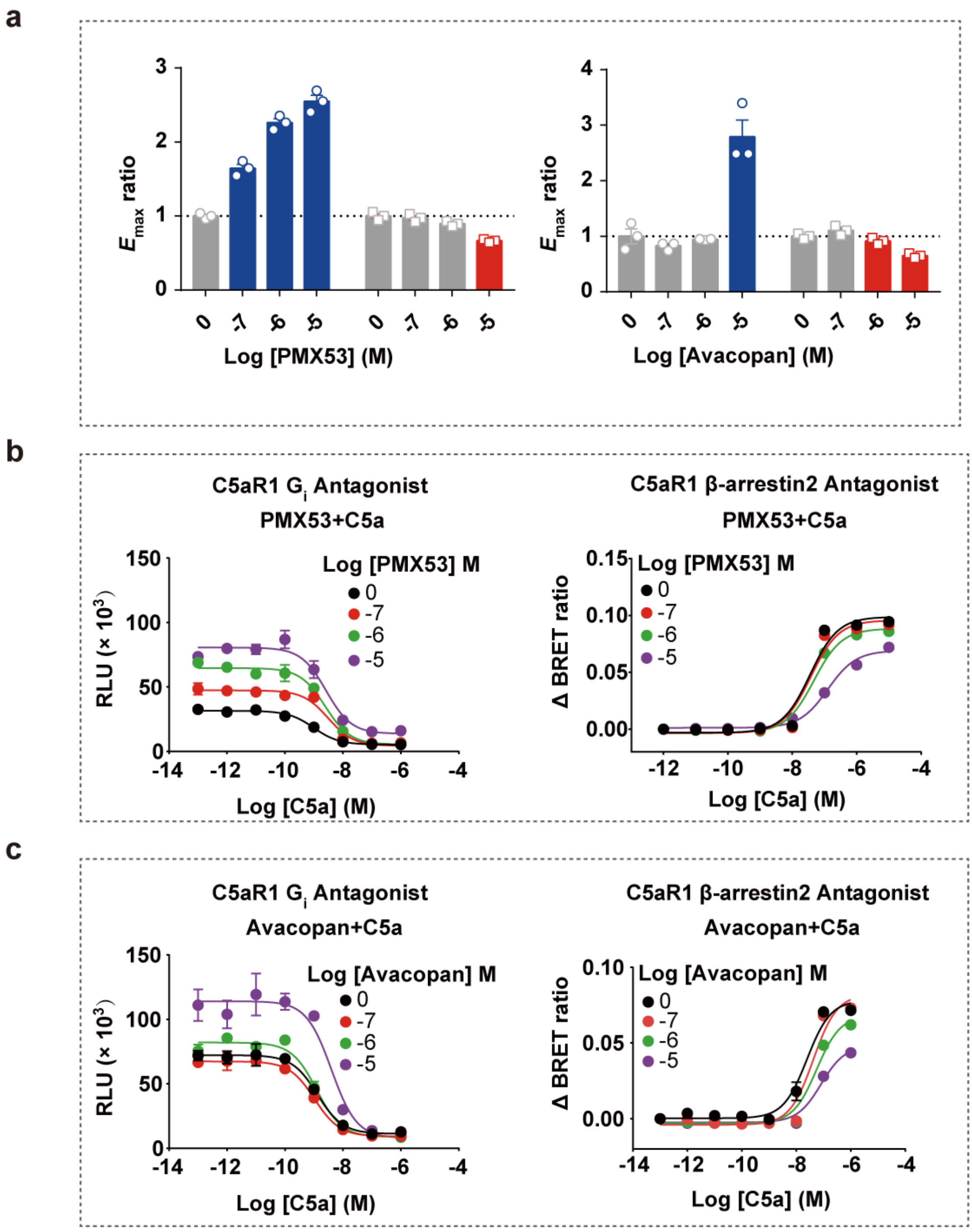

Fig. S8. Antagonism of PMX53 and Avacopan towards C5aR1.

a, The *E*max ratio of PMX53 and Avacopan. Data are presented as the mean ± SEM of three independent experiments performed in triplicate.

b-c, The antagonistic potency of PMX53 (**b**) and Avacopan (**c**) in competition with C5a measuring G_i-protein signaling (left panel) and β-arrestin2 recruitment (right

panel). Data are presented as the mean ± SEM of three independent experiments performed in triplicate.
