# Supplementary material for: Mechanism of activation and biased signaling in complement receptor C5aR1
Source: Cell Res. 2023 Feb 17;33(4):312–24. doi: 10.1038/s41422-023-00779-2 (PMC9937529; doi:10.1038/s41422-023-00779-2)
Supplement: Supplementary file 9 — Supplementary information, Fig. S9 [file 41422_2023_779_MOESM9_ESM.pdf]

Supplementary information, Fig. S9

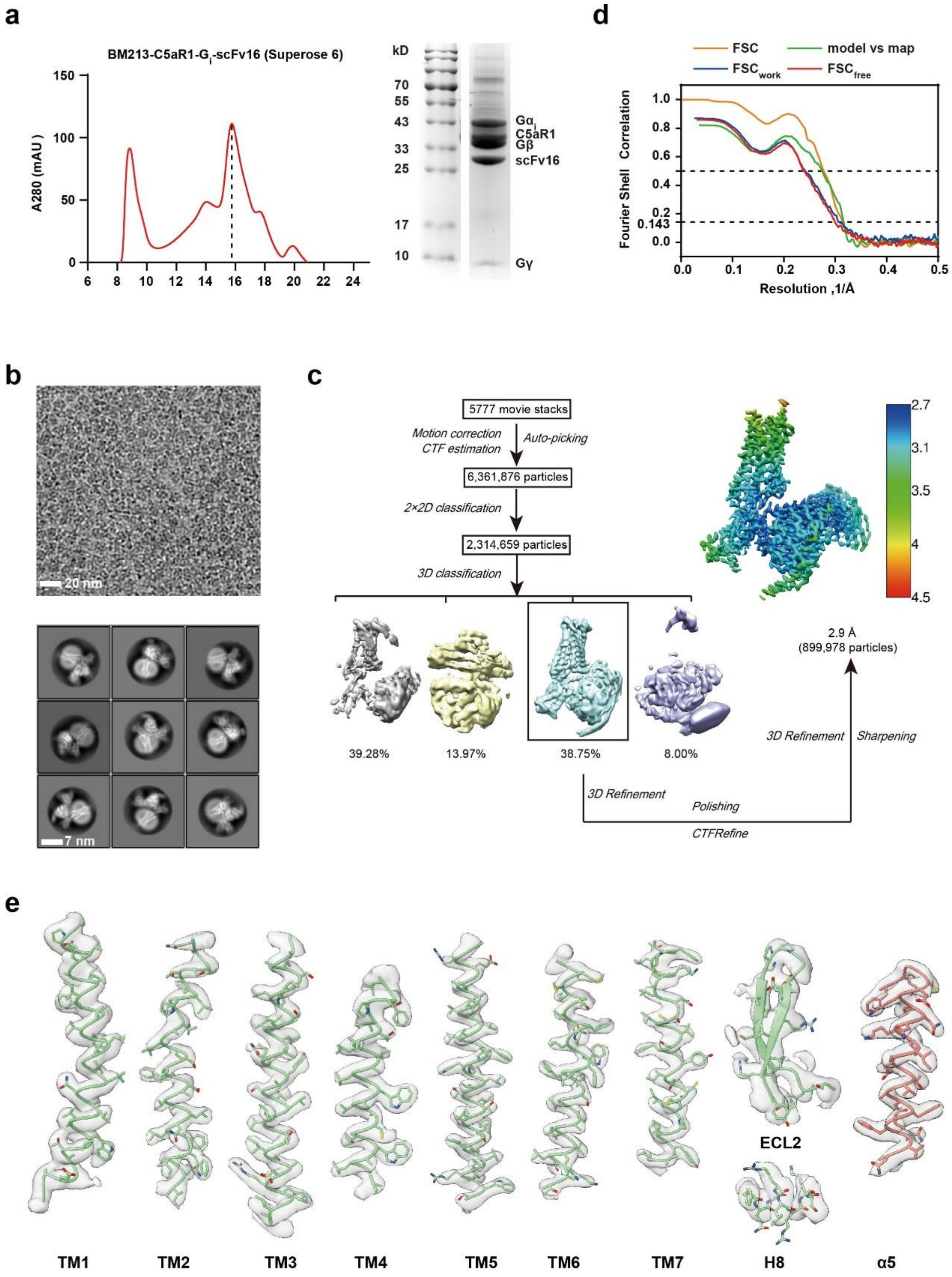

Fig. S9. Single-particle reconstruction of BM213-C5aR1-G_i complex.

a, Representative elution profile of the purified BM213-C5aR1-G_i complex (left panel) and SDS–PAGE of the size-exclusion chromatography peak (right panel)**.**

b, Representative cryo-EM micrographs (upper panel) and 2D classification averages (lower panel) of the BM213-C5aR1-G_i complex.

c, Cryo-EM data processing flowcharts and local resolution for the BM213-C5aR1-G_i complex.
d, Gold-standard FSC curve (orange), the model-vs-map curve (green), FSC_{work} (blue) and FSC_{free} (red) validation curve of $C5a^{pep}$-C5aR1-G_i complex. The marginal gap between the FSC_{work} and FSC_{free} validation curve indicate no over-fitting of the model.
e, Cryo-EM density maps and models for transmembrane helixes TM1-TM7, ECL2 and H8 of C5aR1, as well as the α5 helix of $G\alpha_{i1}$.
