# Supplementary material for: Mechanism of activation and biased signaling in complement receptor C5aR1
Source: Cell Res. 2023 Feb 17;33(4):312–24. doi: 10.1038/s41422-023-00779-2 (PMC9937529; doi:10.1038/s41422-023-00779-2)
Supplement: Supplementary file 10 — Supplementary information, Fig. S10 [file 41422_2023_779_MOESM10_ESM.pdf]

Supplementary information, Fig. S10

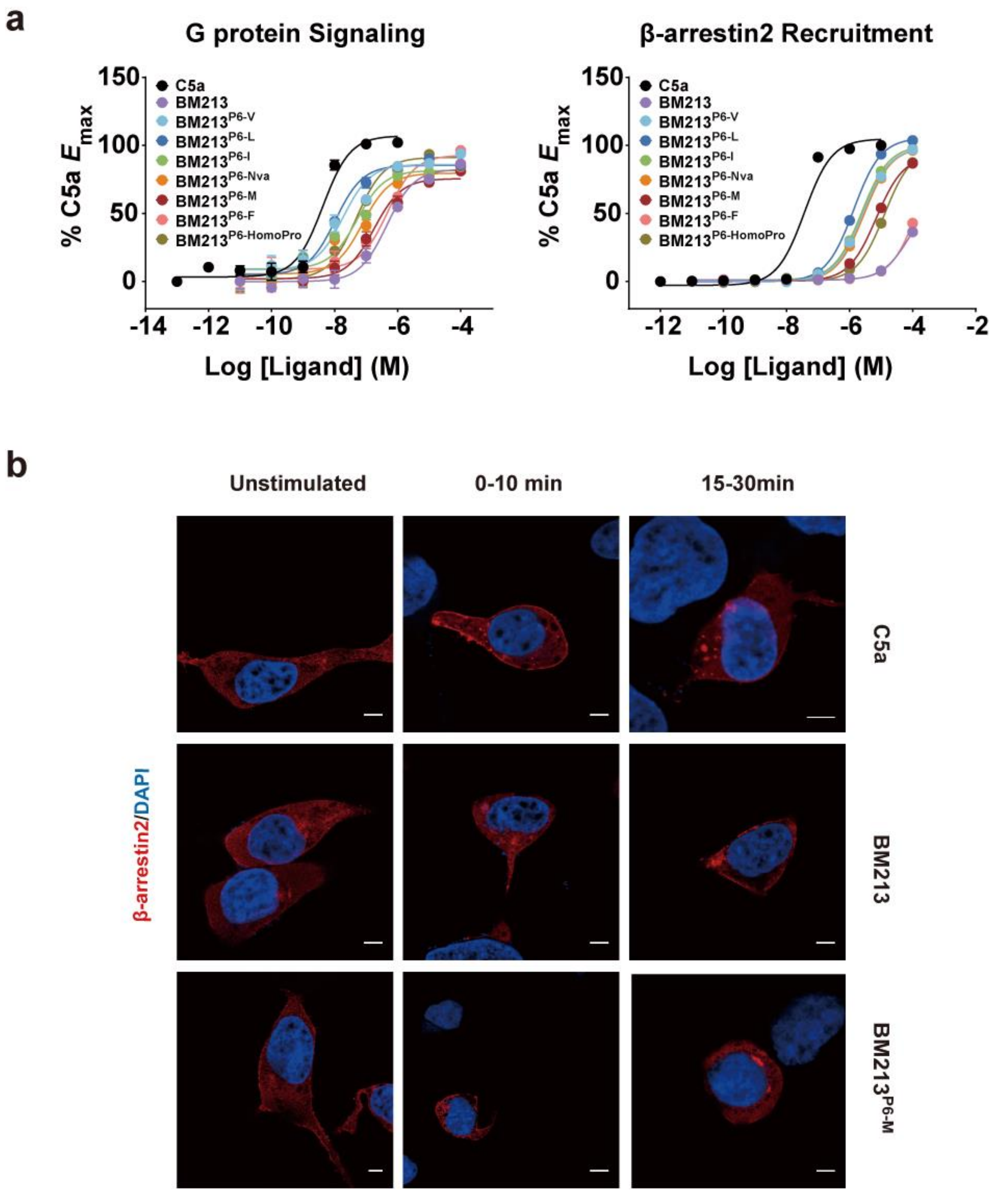

Fig. S10. Effects of mutations at the P6 position of BM213 on G_i and β-arrestin signaling of C5aR1.

a, The signaling properties of BM213 derivates with modification on the P6 position. The G_i protein signaling was detected by G_i protein dissociation assay (left panel) and the β-arrestin2 recruitment was examined by BRET assay (right panel). Data are presented as the mean ± SEM of three independent experiments performed in triplicate.

b, Ligands induced endosomal trafficking of β-arrestin2. HEK293 cells expressing C5aR1-WT and mCherry-β-arrestin2 were stimulated with C5a (100 nM), BM213 (100 μM) and $BM213^{P6\text{-}M}$ (100 μM), and the trafficking patterns of β-arrestin2 were visualized using confocal microscopy at indicated time points. The scale bar represents 5 μm.
