# Supplementary material for: Mechanism of activation and biased signaling in complement receptor C5aR1
Source: Cell Res. 2023 Feb 17;33(4):312–24. doi: 10.1038/s41422-023-00779-2 (PMC9937529; doi:10.1038/s41422-023-00779-2)
Supplement: Supplementary file 11 — Supplementary information, Fig. S11 [file 41422_2023_779_MOESM11_ESM.pdf]

Supplementary information, Fig. S11

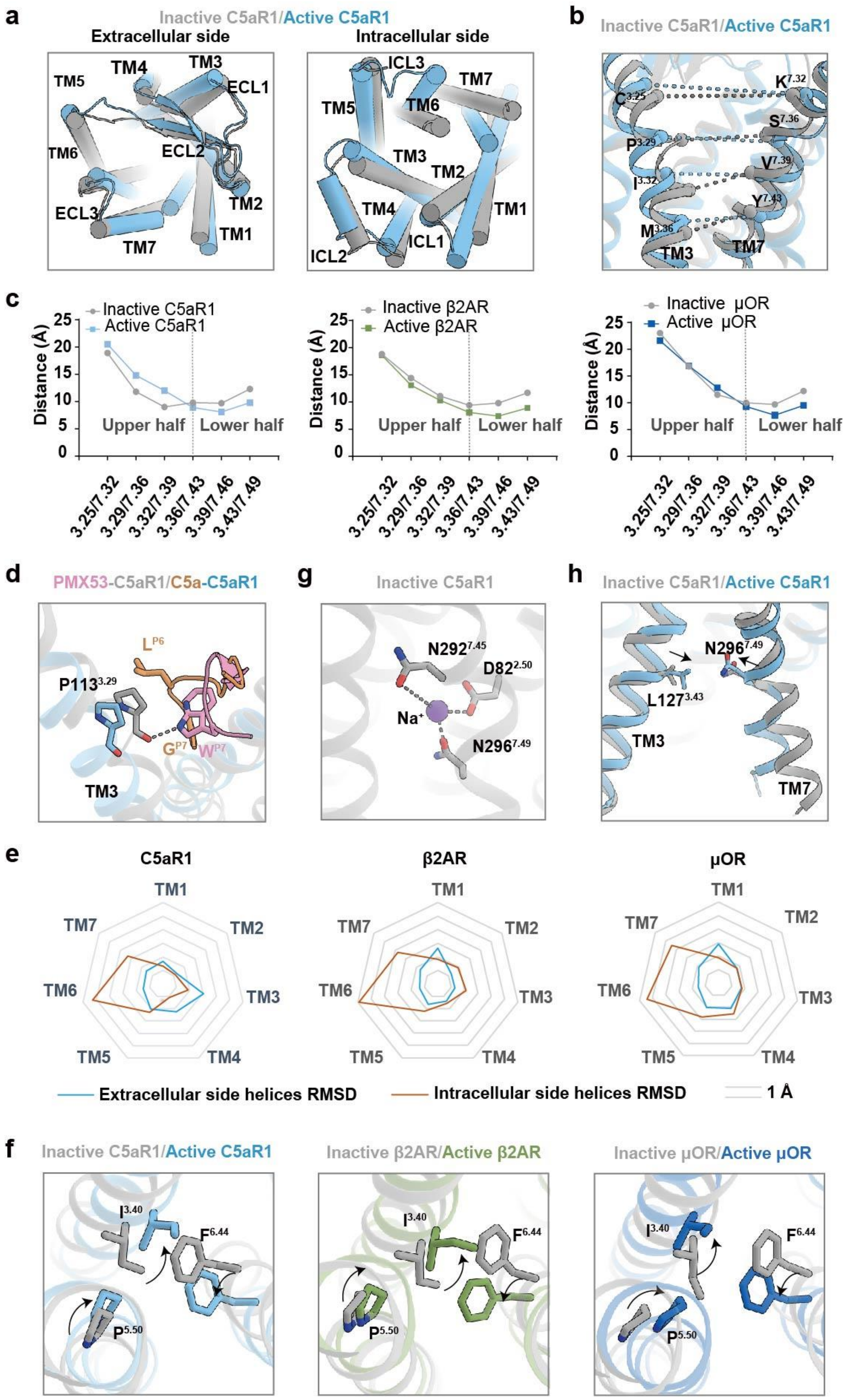

Fig. S11. Structural comparison of C5aR1 in inactive state and active state.

a, Structural superposition of active C5aR1 (C5a-bound) and inactive C5aR1 (PDB: 6C1R) from the extracellular side view (left panel) and intracellular side view (right panel).

b, Structural superposition of active C5aR1 (C5a-bound) and inactive C5aR1 (PDB: 6C1R) from the side view. The extracellular side of TM3 and TM7 undergo an obvious conformational change. The Cα distances of residues between TM3 and TM7 are shown as dashed lines. Inactive C5aR1: gray; active C5aR1: light sky blue.

c, Comparison of Cα distances of residues between TM3 and TM7 of inactive (PDB: 6C1R) and active C5aR1 (left panel), inactive (PDB: 3NY8) and active β2AR (PDB: 3SN6) (middle panel), inactive (PDB: 4DKL) and active μOR (PDB: 6DDE) (right panel).

d, Structural comparison of the antagonist PMX53-bound inactive C5aR1 (PDB: 6C1R) with that of agonist C5a-bound active C5aR1. The indole group of W^{P7} in PMX53 form a H-bond with the main chain of $P113^{3.29}$ to stabilize the inactive information of TM3, instead the L^{P6} and G^{P7} of the agonist C5a disrupted this interaction, allowing the outward displacement of TM3.

e, Comparison of root mean square deviation (rmsd) values of the extracellular side and intracellular side helices between inactive (PDB: 6C1R) and active C5aR1, inactive (PDB: 3NY8) and active β2AR (PDB: 3SN6) (middle panel), inactive (PDB: 4DKL) and active μOR (PDB: 6DDE) (right panel). Residues with Ballesteros–Weinstein numbering 1.46, 2.51, 3.38, 4.50, 5.47, 6.47 and 7.49 were used to divide the transmembrane helices into the extracellular and intracellular side. The rmsd values were calculated in UCSF Chimera.

f, Conformational changes of the conserved "PIF" motif upon C5aR1(left panel), β2AR (middle panel) and μOR (right panel) activation. The conformational changes of residues are shown as arrows.

g, The sodium binding pocked in the inactive state C5aR1 (PDB: 6C1R). The sodium ion was stabilized by $D82^{2.50}$, $N292^{7.45}$ and $N296^{7.49}$.

h, Conformational comparison of $N296^{7.49}$ in TM7 and $L127^{3.43}$ in TM3 between the inactive state C5aR1 and active state C5aR1. Inward displacement of $N296^{7.49}$ and $L127^{3.43}$ was observed upon the activation of C5aR1.
