# Supplementary material for: Mechanism of activation and biased signaling in complement receptor C5aR1
Source: Cell Res. 2023 Feb 17;33(4):312–24. doi: 10.1038/s41422-023-00779-2 (PMC9937529; doi:10.1038/s41422-023-00779-2)
Supplement: Supplementary file 12 — Supplementary information, Fig. S12 [file 41422_2023_779_MOESM12_ESM.pdf]

Supplementary information, Fig. S12

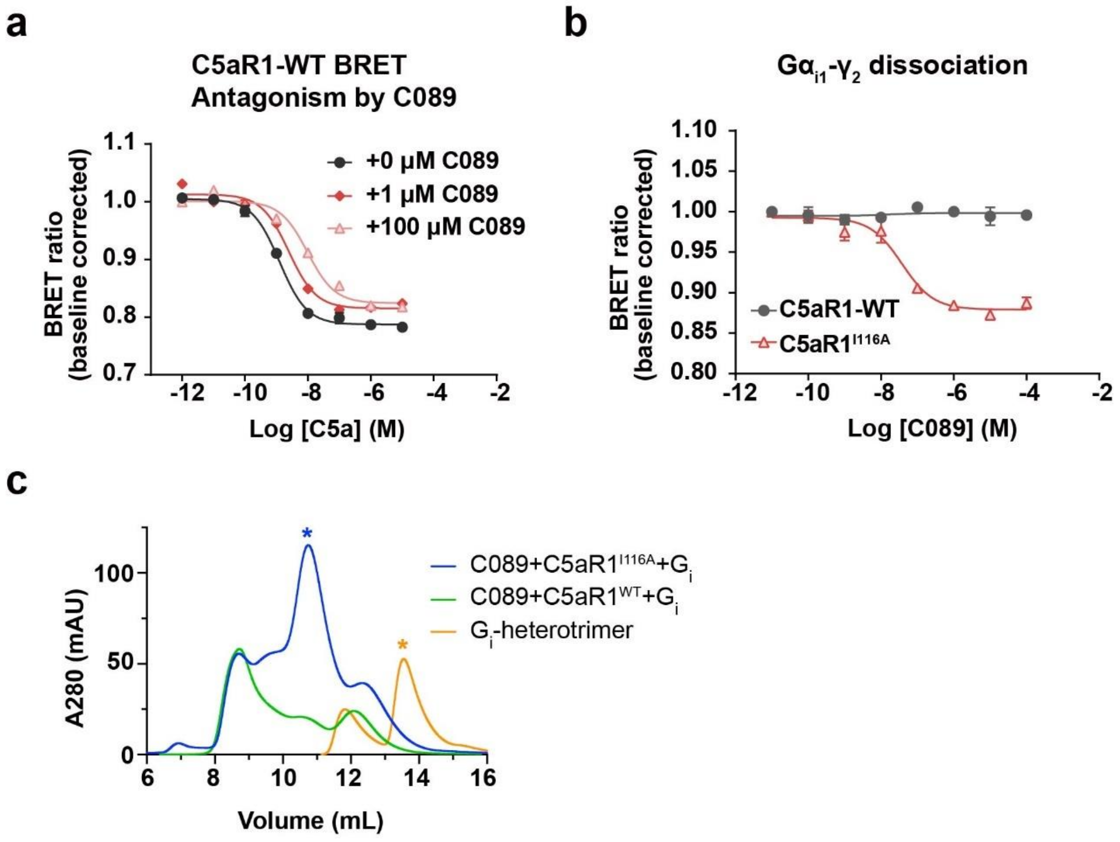

Fig. S12. C089 acts as antagonist for wild-type C5aR1 but an agonist of the C5aR1^{I116A} mutant.

a, BRET data showing C5a-mediated G_i protein dissociation for C5aR1 were antagonized by C089 in a concentration-dependent manner. Data are presented as the mean ± SEM of three independent experiments performed in triplicate.

b, The G_i protein dissociation induced by C089 for C5aR1 and C5aR1^{I116A}, Data are presented as the mean ± SEM of three independent experiments performed in triplicate.

c, Size-exclusion chromatography (SEC) analysis of samples prepared by mixing purified heterotrimeric G_i with either purified C5aR1^{I116A} or C5aR1^{WT} in the presence of C089. The peak of C089-C5aR1-G_i complex is marked with blue asterisk, and the heterotrimeric G_i peak is marked with orange asterisk.
