# Supplementary material for: Mechanism of activation and biased signaling in complement receptor C5aR1
Source: Cell Res. 2023 Feb 17;33(4):312–24. doi: 10.1038/s41422-023-00779-2 (PMC9937529; doi:10.1038/s41422-023-00779-2)
Supplement: Supplementary file 14 — Supplementary information, Fig. S14 [file 41422_2023_779_MOESM14_ESM.pdf]

Supplementary information, Fig. S14

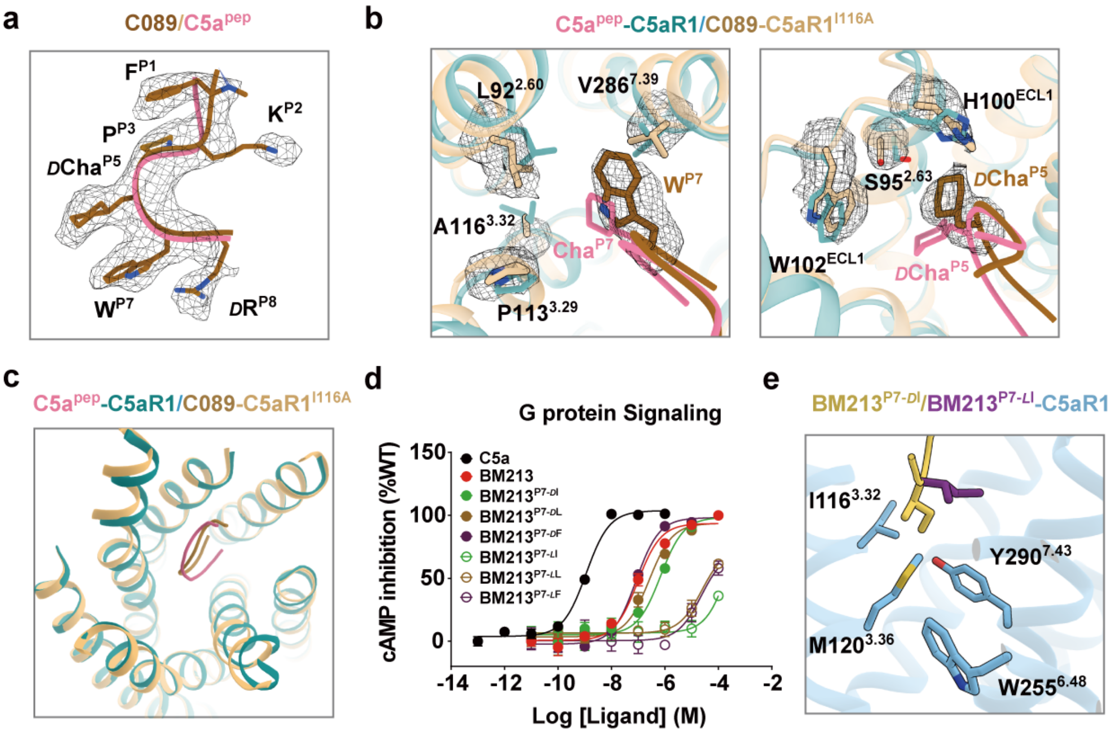

Fig. S14. The structure of C089-bound C5aR1^{I116A}.

a, EM density of C089 in C089-C5aR1^{I116A} complex structure.

b, EM density of W^{P7} (left panel), Cha^{P5} (right panel) and their surrounding residues in C089-C5aR1^{I116A} complex structure, corresponding residues in C5a^{pep}-C5aR1 complex was aligned as a reference.

c, Structural superimposition of C5a^{pep}-C5aR1 with C089-C5aR1^{I116A} showing that the two structures share the similar conformation of TM bundles.

d, BM213 derivates with modification on the P7 position induced G$_i$ protein signaling detected by cAMP inhibition assay. Data are presented as the mean ± SEM of three independent experiments performed in triplicate.

e, Structural comparison of BM213$^{P7-\mathit{DI}}$ and BM213$^{P7-\mathit{LI}}$ in C5aR1. The model of BM213$^{P7-\mathit{DI}}$ and BM213$^{P7-\mathit{LI}}$ were generated and mutated from BM213.
