# Supplementary material for: Mechanism of activation and biased signaling in complement receptor C5aR1
Source: Cell Res. 2023 Feb 17;33(4):312–24. doi: 10.1038/s41422-023-00779-2 (PMC9937529; doi:10.1038/s41422-023-00779-2)
Supplement: Supplementary file 15 — Supplementary information, Fig. S15 [file 41422_2023_779_MOESM15_ESM.pdf]

Supplementary information, Fig. S15

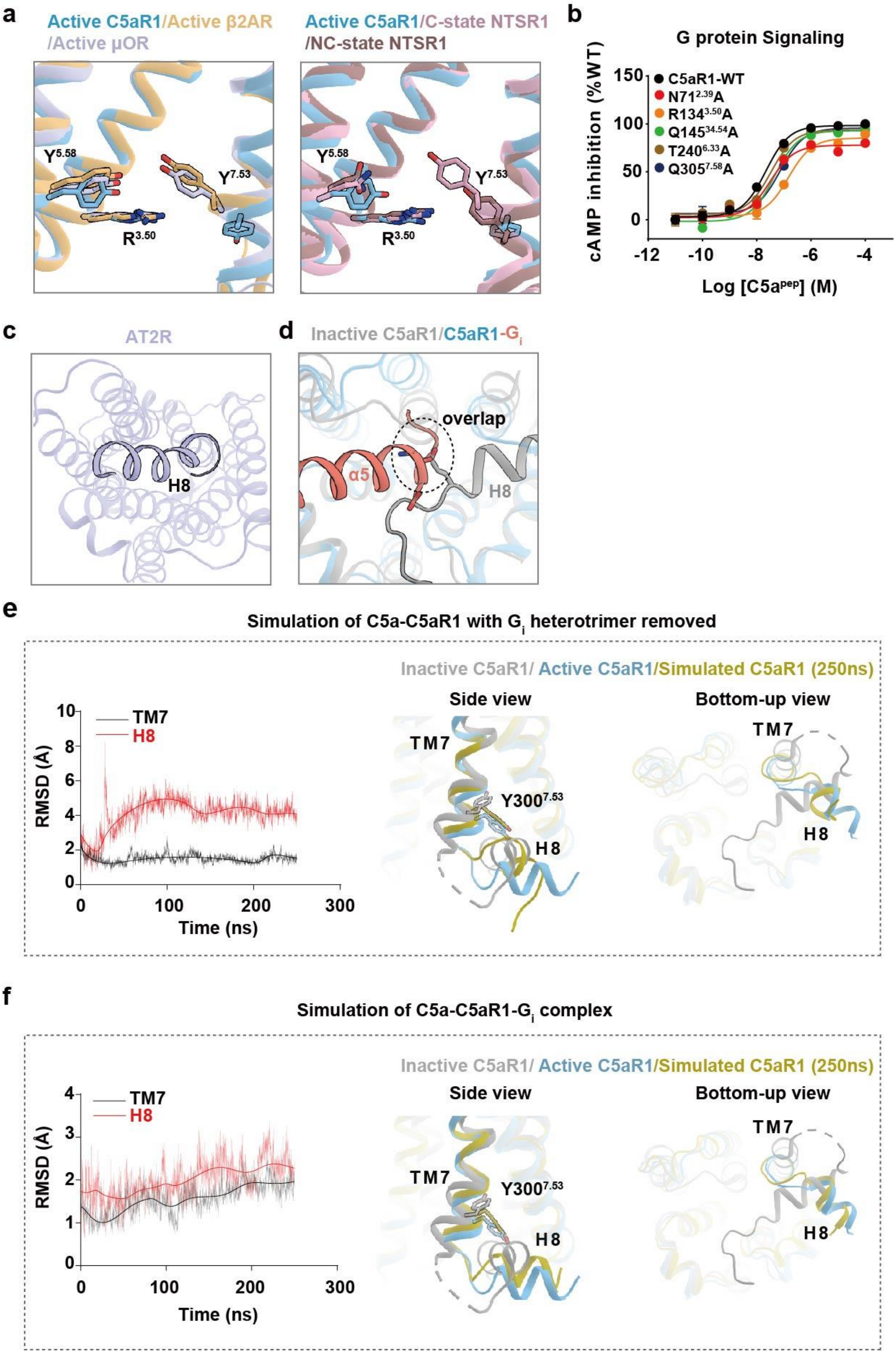

Fig. S15. Noncanonical conformation of TM7 and H8 in C5aR1.

a, Structural superimposition of C5a-bound active C5aR1 with active β2AR (PDB:3SN6), μOR (PDB:6DDE), C-state NTSR1(PDB:6OS9) and NC-state NTSR1 (PDB: 6OSA) showing a unique conformation of $Y^{7.53}$.

b, Representative curve for effects of the $N71^{2.39}A$, $R134^{3.50}A$, $Q145^{34.54}$, $T240^{6.33}A$ and $Q305^{7.58}A$ mutations in C5aR1 on $C5a^{pep}$ induced G protein signaling detected by cAMP inhibition assay. Data are presented as the mean ± SEM of three independent experiments performed in triplicate.

c, The noncanonical conformation of H8 of AT2R (PDB: 5UNG).

d, Structural alignment of inactive C5aR1 and C5aR1-G_i complex showing that the α5 helix of $G\alpha_i$ and H8 in the inactive state occupied overlapped cytoplasmic cavity of C5aR1.

e-f, MD simulation study of C5aR1 using C5a-C5aR1 with G_i heterotrimer removed (**e**) or C5a-C5aR1-G_i complex (**f**) as the starting model. Root Mean Square Deviation (RMSD) of TM7 and H8 relative to the first snapshot during 250 ns classical MD simulation were shown in the left panel. Structural alignment of the simulated C5aR1 at 250 ns with inactive C5aR1 (PDB: 5O9H) and active C5aR1 (cryo-EM structure of C5a-bound C5aR1) were shown in the right panel.
