# Supplementary material for: Mechanism of activation and biased signaling in complement receptor C5aR1
Source: Cell Res. 2023 Feb 17;33(4):312–24. doi: 10.1038/s41422-023-00779-2 (PMC9937529; doi:10.1038/s41422-023-00779-2)
Supplement: Supplementary file 16 — Supplementary information, Fig. S16 [file 41422_2023_779_MOESM16_ESM.pdf]

Supplementary information, Fig. S16

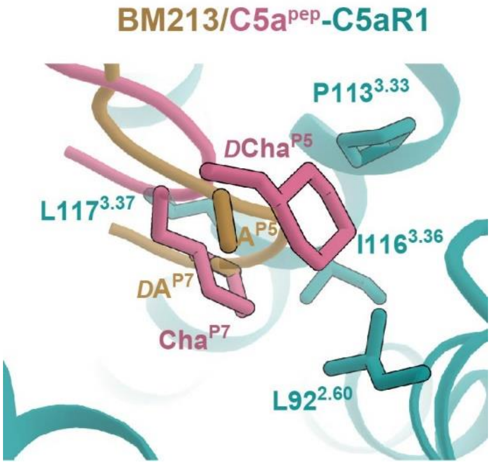

Fig. S16. Structural comparison of BM213 and C5a^{pep} in the orthosteric binding pocket of C5aR1. *D*Cha^{P5} and Cha^{P7} of C5a^{pep} forms more extensive hydrophobic interactions with C5aR1, when compared to BM213.
