# Supplementary material for: Mechanism of activation and biased signaling in complement receptor C5aR1
Source: Cell Res. 2023 Feb 17;33(4):312–24. doi: 10.1038/s41422-023-00779-2 (PMC9937529; doi:10.1038/s41422-023-00779-2)
Supplement: Supplementary file 17 — Supplementary information, Fig. S17 [file 41422_2023_779_MOESM17_ESM.pdf]

Supplementary information, Fig. S17

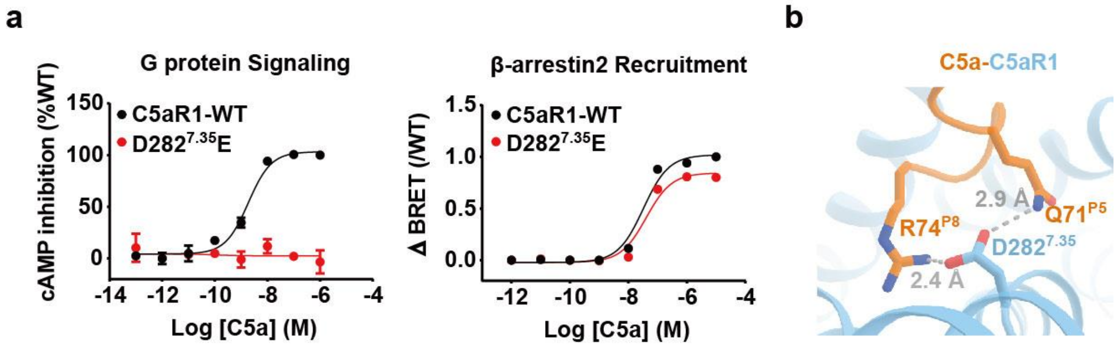

Fig. S17. Effect of D282$^{7.35}$E on C5aR1 induced downstream signaling in response to C5a.

a, The G protein signaling was monitored by cAMP inhibition assay, the β-arrestin2 recruitment was examined by BRET assay. Data are presented as the mean ± SEM of three independent experiments performed in triplicate.

b, The interaction between D282$^{7.35}$ of C5aR1 and C5a. The polar interactions were highlighted with grey dashed lines and the corresponding distance were labeled in the figure.
