# Supplementary material for: Mechanism of activation and biased signaling in complement receptor C5aR1
Source: Cell Res. 2023 Feb 17;33(4):312–24. doi: 10.1038/s41422-023-00779-2 (PMC9937529; doi:10.1038/s41422-023-00779-2)
Supplement: Supplementary file 18 — Supplementary information, Table S1 [file 41422_2023_779_MOESM18_ESM.pdf]

Supplementary information, Table S1

Cryo-EM data collection, refinement and validation statistics of C5aR1-G_i complexes.

Parameters	C5a-C5aR1-G_i complex	C5a^{pep}-C5aR1-G_i complex	BM213-C5aR1-G_i complex	C089-C5aR1^{I116A}-G_i complex
**Data collection and processing**				
Magnification	165,000	165,000	165,000	165,000
Voltage (kV)	300	300	300	300
Electron exposure ($e^-/Å^2$)	66	62	67	63
Defocus range (μm)	-1 to -1.8	-1 to -1.8	-1 to -1.8	-1 to -1.8
Pixel size (Å)	0.85	0.85	0.85	0.85
Symmetry imposed	C1	C1	C1	C1
Initial particle images (no.)	4,588,123	4,329,443	6,361,876	2,955,107
Final particle images (no.)	623,262	191,664	899,978	298,883
Map resolution (Å)	2.9	3.2	2.9	2.8
FSC threshold	0.143	0.143	0.143	0.143
Map resolution range (Å)	2.8-5.0	2.7-6.5	2.7-4.5	2.6-4.5
**Refinement**				
Initial model used (PDB code)	5O9H,7EW3	C5a-C5aR1-G_i	C5a-C5aR1-G_i	C5a-C5aR1-G_i
Model resolution (Å)	3.2	3.6	3.1	3.4
FSC threshold	0.5	0.5	0.5	0.5
Model composition				
Non-hydrogen atoms	9520	8726	8917	8889
Protein residues	1218	1109	1139	1134
Ligands	0	3 (ZAL:1/DAR:2)	2 (DAL: 1/ACE:1)	2 (ZAL:1/DAR:1)
B factors (Å)				
Protein	58.18	51.57	52.84	53.18
Ligand	--	90.06	73.72	74.76
R.m.s. deviations				
Bond lengths (Å)	0.002	0.002	0.002	0.004
Bond angles (°)	0.560	0.544	0.571	1.035
Validation				
MolProbity score	1.36	1.61	1.43	1.49
Clash score	4.89	7.93	6.46	6.99
Poor rotamers (%)	0	0.11	0	0.31
Ramachandran plot				
Favored (%)	97.50	96.96	97.68	97.49
Allowed (%)	2.50	3.04	2.32	2.51
Disallowed (%)	0	0	0	0
