# Supplementary material for: Mechanism of activation and biased signaling in complement receptor C5aR1
Source: Cell Res. 2023 Feb 17;33(4):312–24. doi: 10.1038/s41422-023-00779-2 (PMC9937529; doi:10.1038/s41422-023-00779-2)
Supplement: Supplementary file 19 — Supplementary information, Table S2 [file 41422_2023_779_MOESM19_ESM.pdf]

Supplementary information, Table S2

Summary of C5a and $C5a^{pep}$-induced cAMP inhibition assay of C5aR1. Related to the Methods section "C5aR1-G_i-mediated cAMP inhibition assay".

Mutation	EC_{50} (nM)	Fold	*E*max (%WT)	n	EC_{50} (nM)	Fold	*E*max (%WT)	n	Expression level (%WT)
	**C5a**				**$C5a^{pep}$**				
**C5aR1-WT**	1.87±0.43	1.00	100	6	21.61±2.46	1.00	100	6	100
**$E180^{ECL2}A$**	1.17±0.23	0.63	108.20±4.06	3	-	-	-	3	92.67±9.72
**$Y181^{ECL2}A$**	1.23±0.24	0.66	93.76±3.66	3	-	-	-	3	99.66±16.60
**$F182^{ECL2}A$**	2.29±0.48	1.23	103.00±4.46	3	-	-	-	3	106.13±18.32
**$P183^{ECL2}A$**	1.76±0.44	0.94	97.83±4.61	3	-	-	-	3	79.13±11.46
**$R175^{4.64}A$**	13.05±2.21	6.98	122.80±4.45	3	59.95±13.78	2.77	91.30±3.88	3	127.60±13.64
**$D191^{ECL2}A$**	8.14±1.47	4.36	112.00±4.53	3	316.30±57.61	14.64	91.91±2.96	3	102.13±6.85
**$E199^{5.35}A$**	3.24±0.71	1.73	102.20±4.36	3	129.30±33.29	5.98	94.10±4.53	3	105.11±21.02
**$Y258^{6.51}A$**	7.86±1.53	4.20	110.80±4.81	3	49.84±7.96	2.31	94.56±2.64	3	78.11±6.89
**$D282^{7.35}A$**	12.92±2.54	6.91	110.20±5.01	3	463.90±89.22	21.47	85.40±3.01	3	96.34±5.27
**$S171^{4.60}A$**	4.03±1.33	2.16	90.60±5.41	3	786.40±203.00	36.39	81.09±4.14	3	95.57±5.19
**$D282^{7.35}E$**	ND	ND	ND	3	-	-	-	3	100.17±7.12
**$N71^{2.39}A$**	-	-	-	-	31.50±6.27	1.46	75.46±2.55	3	98.13±18.47
**$R134^{3.50}A$**	-	-	-	-	148.60±26.93	6.88	81.97±2.72	3	107.92±18.31
**$Q145^{34.54}A$**	-	-	-	-	45.08±5.91	2.09	94.61±2.14	3	102.56±8.56
**$T240^{6.33}A$**	-	-	-	-	33.47±6.80	1.55	88.12±3.04	3	105.54±4.70
**$Q305^{7.58}A$**	-	-	-	-	72.97±11.89	3.38	89.34±2.69	3	96.68 ±8.44

ND means no detectable due to the low signal. "–" means no measurement.
