# Supplementary material for: Mechanism of activation and biased signaling in complement receptor C5aR1
Source: Cell Res. 2023 Feb 17;33(4):312–24. doi: 10.1038/s41422-023-00779-2 (PMC9937529; doi:10.1038/s41422-023-00779-2)
Supplement: Supplementary file 20 — Supplementary information Table S3 [file 41422_2023_779_MOESM20_ESM.pdf]

Supplementary information Table S3

Summary of C5a-mediated β-arrestin2 recruitment assay of C5aRs. Related to the Methods section "BRET assay measuring β-arrestin2 recruitment to C5a receptors".

Receptors	EC_{50} (nM)	Fold	*E*max (%WT)	n	Expression level (%WT)
C5aRs			C5a		
**C5aR1-WT**	14.82±2.76	1.00	99.72±3.52	6	100
**$E180^{ECL2}A$**	18.38±2.73	1.24	82.74±2.22	3	108.78±5.51
**$Y181^{ECL2}A$**	14.20±2.50	0.96	117.50±3.80	3	120.10±16.84
**$F182^{ECL2}A$**	31.99±8.06	2.16	102.20±4.20	3	100.51±1.33
**$P183^{ECL2}A$**	16.72±3.80	1.13	75.65±3.12	3	104.90±6.46
**E180- $P183^{ECL2}4A$**	198.60±1.63	13.42	62.94±1.05	3	94.36±2.19
**$D282^{7.35}E$**	40.78±10.59	2.75	86.59±4.06	3	103.21±13.22
**$I91^{2.59}A$**	23.77±4.36	1.60	79.54±2.58	3	88.18±0.41
**$W102^{ECL1}A$**	51.51±18.30	3.48	24.10±1.59	3	98.82±2.05
**$R175^{4.64}A$**	47.84±9.15	3.23	43.97±1.55	3	85.55±1.94
**$D191^{ECL2}A$**	24.48±5.26	1.65	82.18±3.13	3	106.90±1.84
**$E199^{5.35}A$**	38.26±7.94	2.58	90.92±3.38	3	105.50±1.84
**$Y258^{6.51}A$**	55.05±8.07	3.72	43.58±1.20	3	92.45±0.20
**C5aR2-WT**	26.53±5.66	1.00	100	6	100
**$R173^{4.64}A$**	16.18±16.74	0.61	22.78±4.29	3	93.00±11.43
**$D189^{ECL2}A$**	ND	ND	ND	3	92.19±2.93
**$E197^{5.35}A$**	19.54±7.79	0.74	85.58±6.32	3	90.16±0.72
**$Y249^{6.51}A$**	ND	ND	ND	3	111.16±1.05

ND means no detectable due to the low signal.
