# Supplementary material for: Mechanism of activation and biased signaling in complement receptor C5aR1
Source: Cell Res. 2023 Feb 17;33(4):312–24. doi: 10.1038/s41422-023-00779-2 (PMC9937529; doi:10.1038/s41422-023-00779-2)
Supplement: Supplementary file 21 — Supplementary information, Table S4 [file 41422_2023_779_MOESM21_ESM.pdf]

Supplementary information, Table S4

Summary of C5a^{pep} and BM213-mediated β-arrestin2 recruitment assay of C5aR1.

**Mutation**	**EC$_{50}$ (μM)**	**Fold**	***E*max (%WT)**	**n**	**EC$_{50}$ (μM)**	**Fold**	***E*max (%WT)**	**n**	**Expression level (%WT)**
		**C5a^{pep}**					**BM213**		
**C5aR1-WT**	1.99±0.11	1.00	100	6	59.95±5.68	1.00	100	6	100
**I91$^{2.59}$A**	ND	ND	ND	3	-	-	-	3	88.18±0.41
**W102^{ECL1}A**	ND	ND	ND	3	ND	ND	ND	3	98.82±2.048
**I116$^{3.32}$F**	0.31±0.07	0.16	106.40±4.43	3	13.33±1.35	0.22	136.80±2.85	3	108.95±7.45

ND means no detectable due to the low signal. "–" means no measurement.
