# Supplementary material for: Mechanism of activation and biased signaling in complement receptor C5aR1
Source: Cell Res. 2023 Feb 17;33(4):312–24. doi: 10.1038/s41422-023-00779-2 (PMC9937529; doi:10.1038/s41422-023-00779-2)
Supplement: Supplementary file 22 — Supplementary information, Table S5 [file 41422_2023_779_MOESM22_ESM.pdf]

Supplementary information, Table S5

Summary of C5a, BM213 and BM213 derivates with modification on the P6 position induced G protein signaling and β-arrestin2 recruitment of C5aR1.

Agonist	EC_{50}(nM)	*E*max (%C5a)	n	EC_{50}(μM)	*E*max (%C5a)	n
	G protein signaling			β-arrestin2 recruitment		
**C5a**	3.67±0.46	100	3	0.04±0.01	100	3
**BM213**	512.60±55.03	89.49±1.79	3	82.50±13.57	36.41±0.84	3
**$BM213^{P6-V}$**	35.53±4.23	84.95±1.72	3	2.59±0.11	96.92±3.09	3
**$BM213^{P6-L}$**	16.72±1.74	83.62±1.55	3	1.35±0.03	103.60±1.65	3
**$BM213^{P6-I}$**	52.87±4.06	84.79±1.14	3	2.39±0.08	97.48±2.16	3
**$BM213^{P6-Nva}$**	90.22±10.02	89.29±1.83	3	2.88±0.11	96.36±2.52	3
**$BM213^{P6-M}$**	159.00±15.77	93.14±1.68	3	6.99±0.38	86.83±2.48	3
**$BM213^{P6-F}$**	455.10±34.91	103.40±1.45	3	154.90±59.01	42.86±2.38	3
**$BM213^{P6-HomoPro}$**	92.37±13.30	90.18±2.40	3	12.80±0.46	87.39±1.91	3
